# A machine learning approach to predict C–H bond activation on dual-site doped graphene catalysts

**DOI:** 10.1039/d6ra03770d

**Published:** 2026-09-21

**Authors:** Amrish Kumar, Iqra Ahangar, Shantanu Suryakant Sontakke, Manojkumar Ramteke, Tuhin S. Khan, M. Ali Haider

**Affiliations:** a Renewable Energy and Chemicals Lab, Department of Chemical Engineering, Indian Institute of Technology Delhi Hauz Khas New Delhi 110016 India haider@iitd.ac.in; b Engineering Technology and Science Division, Department of Chemical Engineering, Higher College of Technology Abu Dhabi 25026 United Arab Emirates; c Department of Chemical Engineering, Indian Institute of Technology Delhi Hauz Khas New Delhi India; d Climate Changes and Data Science Division, CSIR-Indian Institute of Petroleum Mohkampur Dehradun 248005 India tuhin.khan@csir.res.in; e IIT Delhi-Abu Dhabi Khalifa City B Abu Dhabi 20010 United Arab Emirates

## Abstract

The catalytic performance of graphene-based dual-metal-site catalysts (DMSCs) for the nonoxidative coupling of methane (NOCM) is systematically investigated using machine learning (ML)-assisted DFT calculations. To obtain insights into the methane C–H bond activation, several ML algorithms are used to predict adsorption energies (
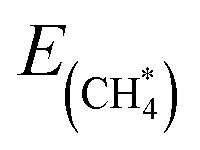
 and 
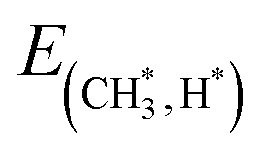
) and activation barriers (*E*_a_) at each step. Initially, DFT-evaluated energies for the first C–H activation step of NOCM are used as the target variable for training various ML models. In addition, several readily available features associated with transition-metal-doped graphene are used as input features to construct a dataset, which is then used by ML models to predict the catalytic activity of DMSCs. All models are validated by performing 100 random splits of the training and test data to avoid training bias. Model validation, performed through 100 random train-test splits, identified random forest regression (RFR) and extra tree regression (ETR) as the most reliable predictors, yielding test errors of 0.33 eV for 
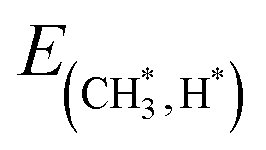
 and 0.27 eV for *E*_a_. Beyond the initial C–H activation, the study explores the C–C coupling pathway leading to C_2_ hydrocarbon formation. Among the DMSC configurations examined, Fe/Ni and Fe/Cu exhibited the lowest coupling barriers, demonstrating their superior catalytic activity. This study demonstrates that ML models can be effectively used for catalyst screening, providing promising descriptors from input features that can accelerate high-throughput screening and rational design of efficient catalysts for methane activation and coupling reactions.

## Abstract 2

A machine learning framework trained on DFT-derived descriptors predicts C–H activation and adsorption energies across graphene dual-metal-site catalysts, accelerating the screening of nonoxidative methane coupling catalysts.

## Graphical abstract

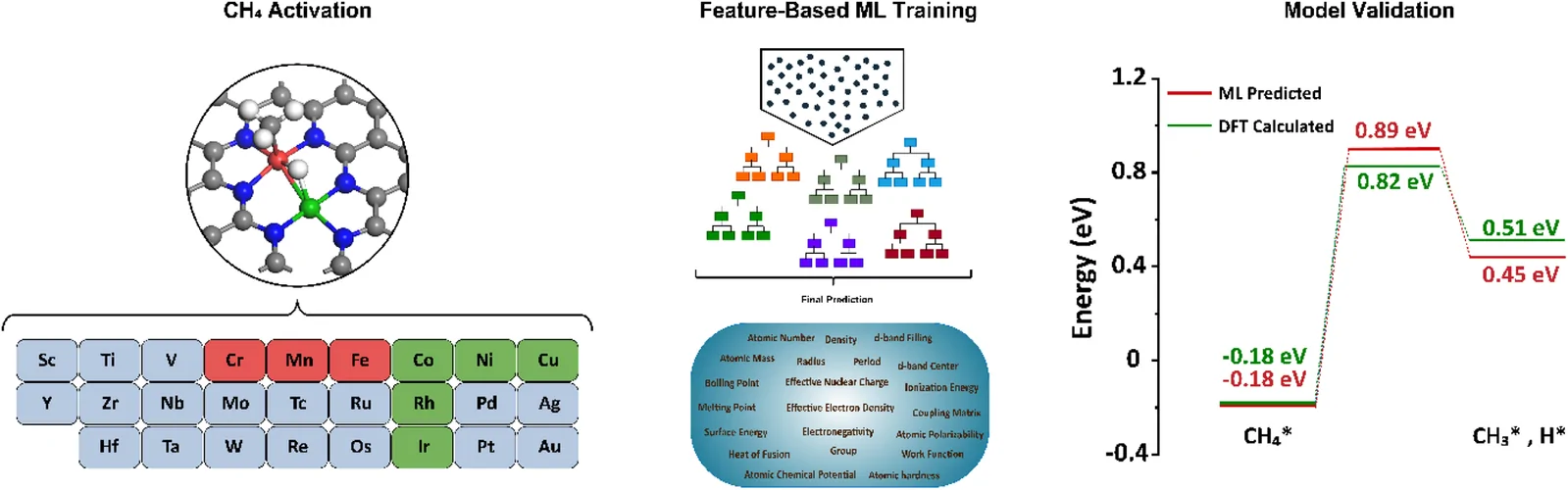

## Introduction

Methane is widely available in natural and shale gas reservoirs, making it a new hydrocarbon source that drives an ever-increasing demand for its conversion into value-added chemicals.^1^ When released into the atmosphere, methane is a hazardous greenhouse gas with a GHG potential 25 times that of CO_2_. Therefore, it is crucial to find efficient catalysts to convert methane *via* both indirect and direct routes. Using the standard indirect approach, methane is first converted into syngas *via* steam reforming and then into value-added chemicals, making this process energy-intensive. On the other hand, employing oxidative and nonoxidative coupling of methane (OCM and NOCM, respectively) to directly dehydrogenate CH_4_ into C_2^+^_ hydrocarbons (such as olefins and aromatics) is a promising pathway.^2^ NOCM is a more desirable approach as it significantly reduces the energy cost by bypassing the process of separating CO and CO_2_, the overoxidized species found in OCM, and produces valuable H_2_ as a by-product.

Single-atom catalysts (SACs) and dual-metal-site catalysts (DMSCs), due to their maximum atom-utilization efficiency, have emerged as new topics of research in the field of heterogeneous catalysis. One of the earliest experimental studies that highlighted the potential of SACs used 0.5 wt% Fe embedded in an SiO_2_ matrix for the nonoxidative conversion of CH_4_, exhibiting high selectivity (>99%) and conversion (∼48%). It was also observed that no significant deactivation of the catalyst occurred even after 60 h at 1293 K.^3^ Then, recently, a nanoceria-supported atomic Pt catalyst was used for NOCM, yielding a 14.4% methane conversion rate and a 74.6% C_2_ selectivity.^4^ Ta_8_O_2_^+^ clusters have also been reported to have dual functionality as C–C coupling occurs at dual Ta sites, similar to nanoceria-supported atomic Pt catalysts, in which both ceria and Pt participate in the activation of CH_4_, resulting in good selectivity.^5^ Therefore, these results indicate that bifunctional catalysts represent promising candidates and need to be explored. Although fabricating them is a challenging task, recently, graphene-based DMSCs have successfully been synthesized by embedding Fe–Co on N-coordinated graphene, exhibiting dual active sites for the acidic oxygen reduction reaction (ORR). In this study, the O_2_ and OOH dissociation barriers of DMSCs were found to be much lower than those of graphene-based SACs.^6^ This indicates that the catalytic activity of transition metals in highly diluted forms can be enhanced in the presence of graphene-based supports.

In this regard, several density functional theory (DFT) studies using N-coordinated graphene-based SACs and DMSCs for CH_4_ conversion have been reported recently. To fabricate a graphene-based transition metal catalyst, Wu and Gates^7^ embedded a single Fe atom in graphene with different configurations by varying the defects from a single valency (SV) to a double valency (DV) and by varying the nitrogen (1–4 N atoms) doping surrounding the Fe atom. The Fe/SV-N123 catalyst configuration (*i.e.*, Fe embedded in single-valence-deficient graphene and surrounded by N doping on all sides) was reported to be the best candidate, with a minimum energy barrier of 0.33 eV for methane activation. Yuan *et al.*^8^ proposed a graphene-based SAC for the oxidative conversion of CH_4_ to methanol and reported a graphene-supported CoO single-atom catalyst exhibiting high reactivity (low CH_4_ activation energy of 0.7 eV) and high selectivity for methanol over other possible methyl and carbene products. To facilitate NOCM, two C–H dissociation steps need to take place simultaneously using two CH_4_ molecules. Therefore, the presence of dual metal active sites is of paramount importance, as CH_4_ activation represents only the first step in methane conversion, leading to a wide range of subsequent reactions originating from C–H bond dissociation during both methane activation and dehydration processes.^9^ Dual sites on a catalyst are highly favorable for coupling reactions as they increase the probability of coupling of the two carbons, which can result in the production of C_2_ and higher hydrocarbons. In this regard, Huang *et al.* conducted a detailed study using various configurations of Fe and Co doped on N-coordinated graphene (Fe–N–C, Co–N–C, Fe_2_–N–C, FeCo–N–C, and Co_2_–N–C) for NOCM. They discovered that compared to SACs, the Co_2_–N–C DMSC was stable at high temperatures, showing good selectivity towards ethane, as demonstrated by microkinetic modelling (MKM) at 1200 K.^10^ In addition, Wu and coworkers also reported the superiority of DMSCs over SACs for CH_4_ activation in their study.^11^

In an effort to shift from precious-metal catalysts to nonprecious ones, Wang *et al.*^12^ successfully fabricated and demonstrated the utility of the Fe–Co DMSC as a potential commercial Pt/C catalyst for the oxygen reduction reaction (ORR). They reported that the DMSC exhibited superior performance to the traditional Pt/C catalyst, with stable operation for 100 h in an H_2_/air fuel cell. In yet another study, Lu and coworkers^13^ developed and tested the Zn–Co DMSC as a cathode catalyst for a Zn–air battery, confirming its exceptional ORR activity and operational stability. Demonstrating the diversity of DMSCs in catalyzing reactions, Ren *et al.*^14^ synthesized the Ni–Fe DMSC to assess its efficacy in the CO_2_ reduction reaction (CO_2_RR), reporting a faradaic efficiency of 98%, high selectivity, and minimal performance degradation even after continuous 30 h operation. In a separate experimental study, a DMSC based on Zn–Fe was demonstrated as a viable electrocatalyst for NH_3_ synthesis *via* the nitrogen reduction reaction (NRR) by Zhang *et al.*,^15^ showing stable operation and a high NH_3_ production rate under ambient reaction conditions. Strengthening the transition to green chemistry, the Fe–Co-based DMSC has shown promise as a catalyst for Fenton-like reactions, offering a sustainable approach to wastewater treatment by eliminating refractory organic contaminants. This was substantiated by Yang *et al.*^16^ through a bisphenol A degradation reaction, demonstrating an outstanding turnover frequency of 60.62/min and the remarkable stability of Fe–Co DMSC, with negligible degradation even after 7 consecutive experimental runs. Further, Zhang and coworkers^17^ reported an efficient alternative to the Haber–Bosch process using the Fe–Ni DMSC for urea synthesis. They observed a high urea yield rate of 20.2 mmol h^−1^ g^−1^ and a faradaic efficiency of 17.8%, outperforming other noble-metal electrocatalysts. These studies present some of the contributions of DMSCs to ongoing efforts to transition from precious-metal catalysts to nonprecious alternatives across various applications, demonstrating the potential of DMSCs in heterogeneous catalysis.

**Fig. 1:**
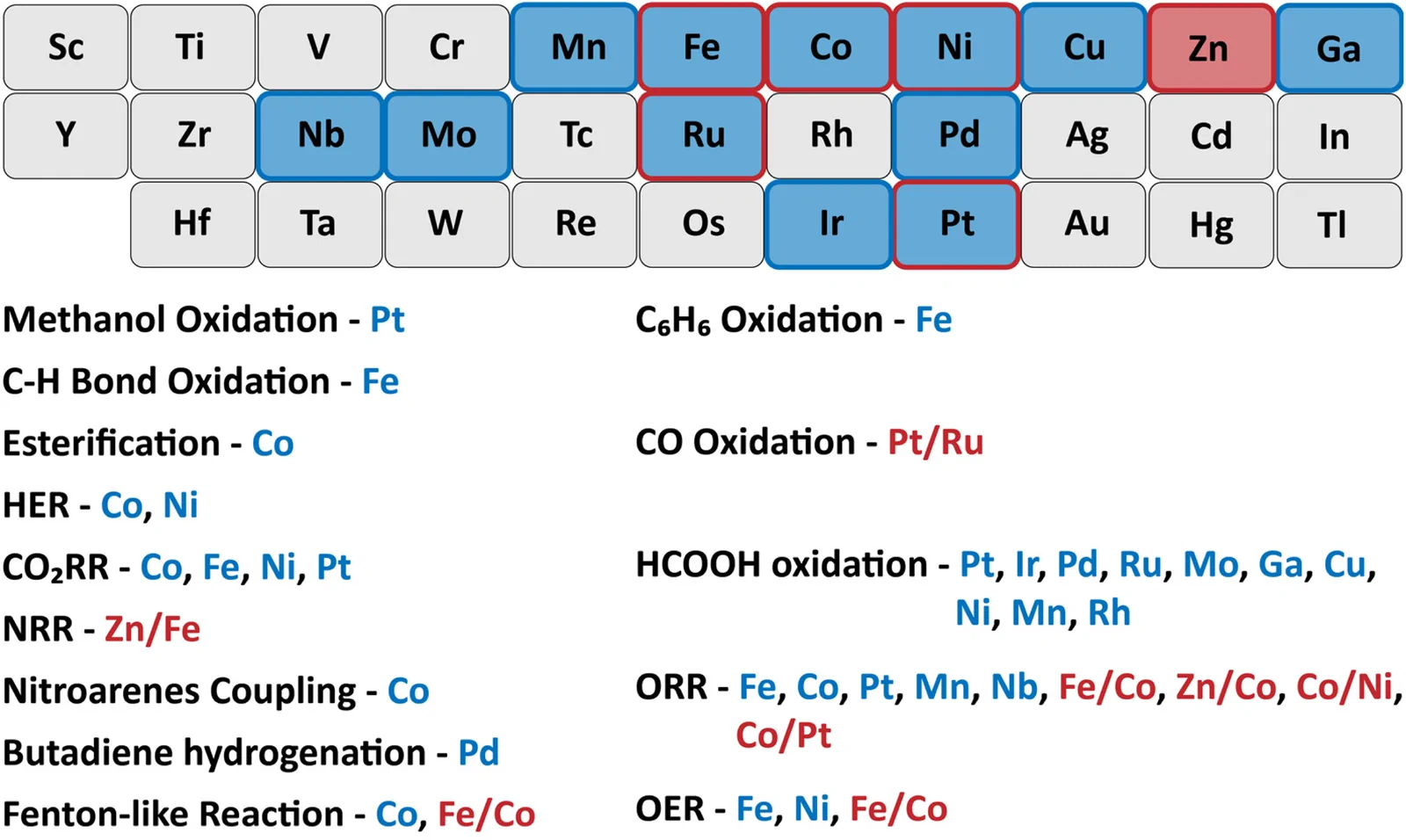
Representation of the experimentally validated single-metal-site catalysts (SMSCs) and dual-metal-site catalysts (DMSCs) for various reactions.

Inspired by these recent developments, as shown in Fig. 1, we have built a dataset of all possible configurations using transition metals to fabricate N-coordinated graphene-based DMSCs to enhance our understanding of possible dual-active-site catalysts for the NOCM reaction *via* a DFT-ML approach. In this study, the initial C–H activation step involved in NOCM is explored extensively because this C–H bond cleavage represents the highest thermodynamic and kinetic barrier, making it the critical rate-limiting step in methane activation.^18–21^ Instead of predicting atomic/molecular or intermediate adsorption energies, we have predicted the activation energy (*E*_a_) by incorporating periodic table properties, along with d-band properties associated with the metals at the dual sites of the catalyst. As transition-state calculations using DFT are time-consuming, we aim to significantly reduce computational costs while illuminating the nonlinear relationship between the material structure and properties.

## Methodology

**Fig. 2:**
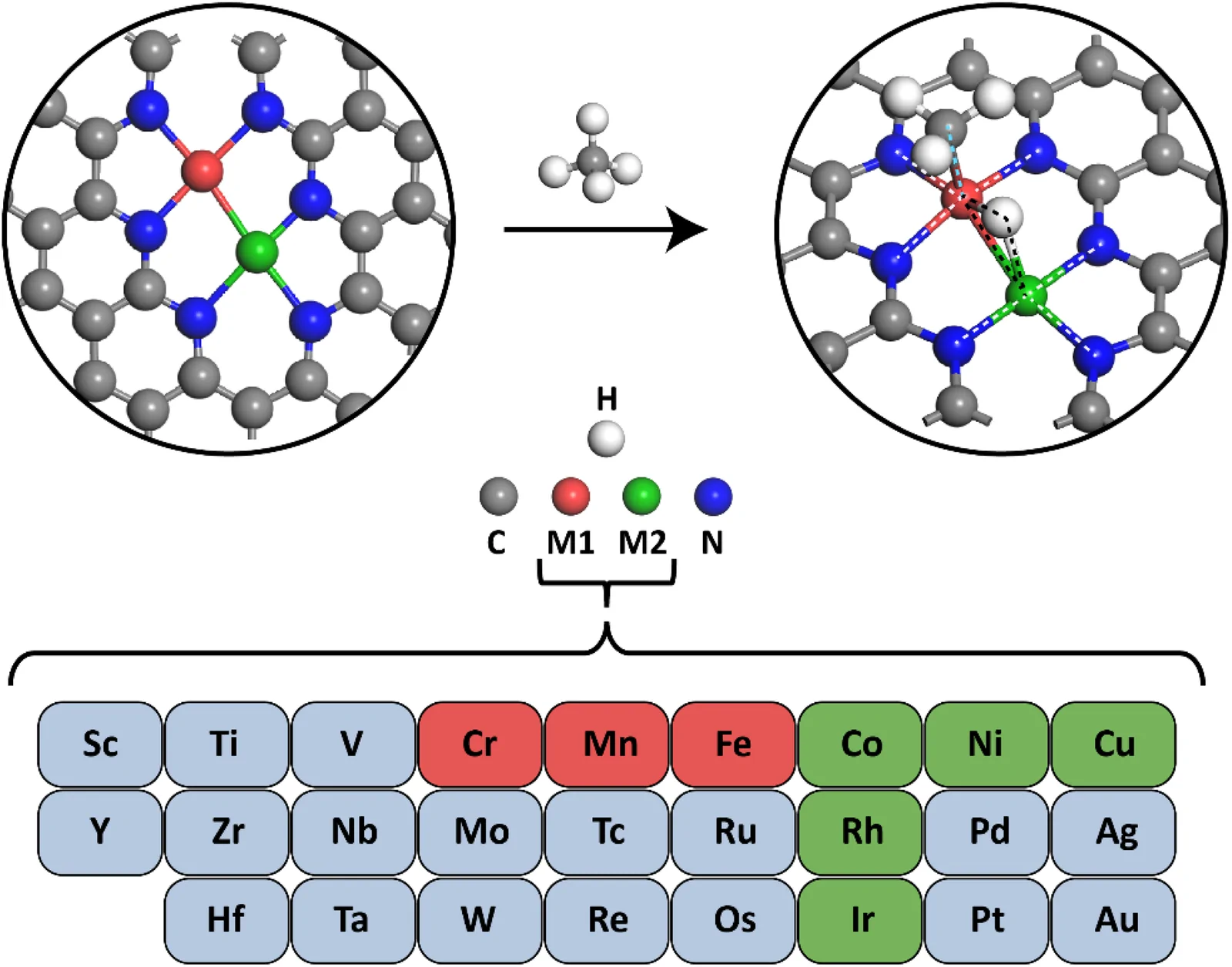
Model of a dual-metal-site catalyst (DMSC) on nitrogen-doped graphene, and the selection of transition metals from the periodic table to construct different DMSC configurations for methane activation.

A single graphene layer of size (4 × 5) was cleaved from the graphite structure available in the Materials Studio library. This layer, representing the catalyst surface, consisted of 40 carbon atoms and was modelled with a vacuum spacing of 15 Å along the *z*-direction to eliminate interlayer interactions. To construct the DMSC, two different transition metal atoms were introduced, along with six nitrogen atoms, by substituting a total of nine carbon atoms in the graphene framework, as illustrated in Fig. 2. The transition metals were systematically selected from the 3d, 4d, and 5d series of the periodic table to investigate both period and group effects on catalytic performance. Specifically, M1 was chosen from Cr, Mn, or Fe (highlighted in red), while M2 (highlighted in green) was chosen from Co, Ni, and Cu (3d series) and Rh and Ir (group 9 series). The catalyst surface in the supercell finally consisted of 39 atoms.^10^

We performed spin-polarised DFT calculations for these DMSCs using the Vienna *ab initio* simulation package (VASP) version 6.2.0.^22^ The core electrons were treated with projector-augmented wave (PAW)^23^ pseudopotentials, while the valence electron wavefunctions were estimated using a plane-wave basis set truncated at 396 eV. To validate this cutoff energy, preliminary benchmarking was conducted against the standard 400 eV criterion, as used by Huang *et al.*^10^ Both settings yielded identical energetic and structural profiles. Consequently, given the computationally expensive nature of generating a large-scale machine learning dataset, 396 eV was adopted to optimally balance high-throughput efficiency with rigorous chemical accuracy. In the reciprocal space, the Monkhorst–Pack scheme was used to calculate the irreducible Brillouin zone with a *k*-point mesh of 2 × 2 × 1. Further, to account for electron self-interactions, a generalized gradient approximation was used with the Perdew–Burke–Ernzerhof (PBE)^24^ exchange–correlation functional, as proposed by Huang *et al.*^10^ for DMSCs. To correct for the dispersive van der Waals interactions between the adsorbates, the DFT-D3 method with Becke–Jonson damping was used.^25^ The energy and force of the optimized structure were converged to 10^−6^ eV and 0.05 eV Å^−1^, respectively. Following geometry optimization of the adsorbate structures, the binding energy was estimated as the difference between the energy of the surface-adsorbed structure and the sum of the gas-phase adsorbate and bare-surface energies.

The reactant and product state structures were connected *via* a linear interpolation of 16 images along the reaction coordinate, and the minimum energy path (MEP) connecting the two was converged using the nudged elastic band method.^26^ The final structures of the transition state were estimated using the climbing image nudged elastic band (CI-NEB) method, as proposed by Henkelman *et al.*,^26^ in which the structure exhibiting the maximum energy on the MEP is allowed to converge to an energy maximum condition that shows an imaginary normal mode frequency along the reaction coordinate and a positive real frequency in all other directions. The energy and force convergence criteria in NEB calculations were set to 10^−6^ eV and 0.05 eV Å^−1^, respectively. The *E*_a_ in the C–H bond dissociation step of methane was estimated as the difference between the transition state (TS) energy of the C–H activation and the initial state (IS) adsorption energy 
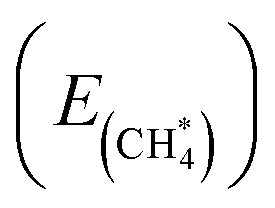
 of the reactant. The reaction energy of the elementary reaction step was estimated as the difference between the final state (FS) adsorption energy 
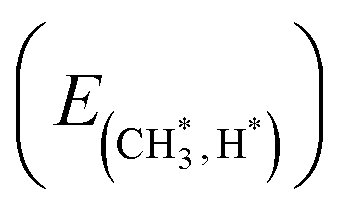
 and the IS energy. The electronic density of states (DOS) was calculated using the same *k*-points, and the d-band centre was estimated using the VASPKIT program.^27^

**Fig. 3:**
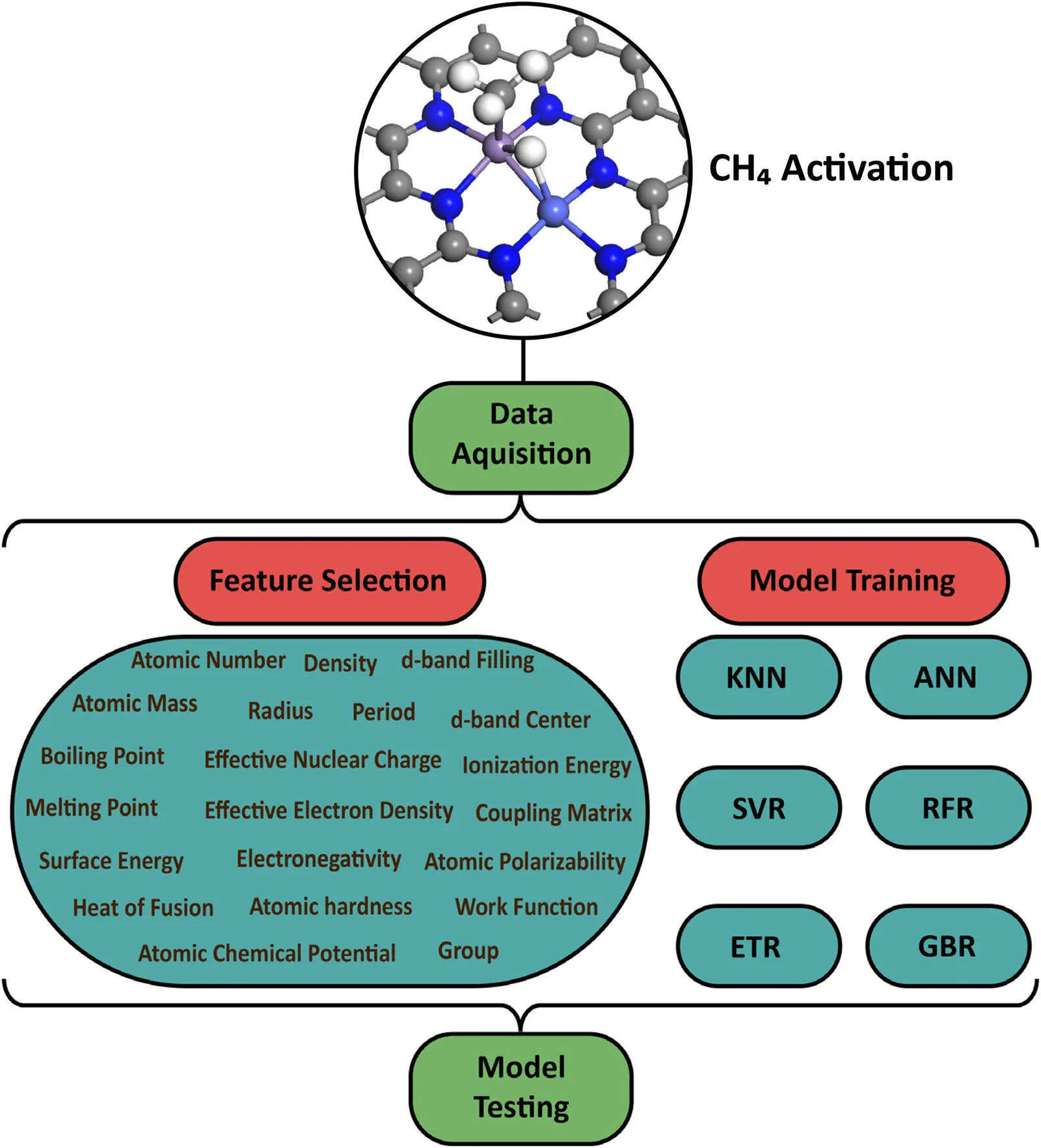
Workflow for predicting C–H activation on N-coordinated graphene-based DMSCs using the ML approach.

The approach of this study was delineated into 3 tasks (*viz.*, data acquisition and pre-processing, ML model development, and model performance analysis), as presented in Fig. 3. The foremost task consisted of the generation of the data encompassing 
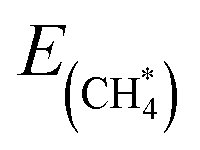
, *E*_a_, and 
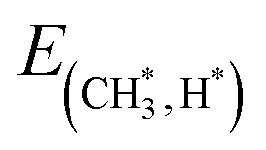
 calculations from DFT for methane activation over all DMSC surface combinations. 
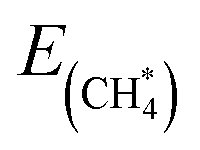
 was defined as “
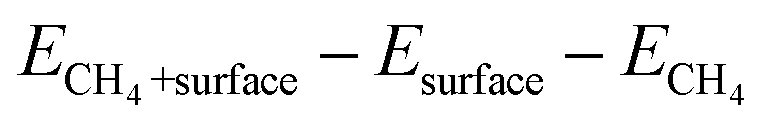
”, and 
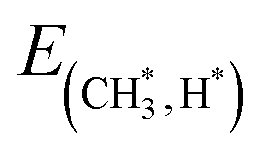
 was defined as “
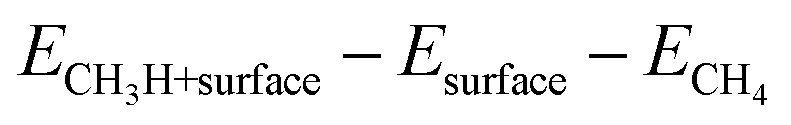
”, where *E*_CH_4__ is the energy of the optimized structure with CH_4_ adsorbed on the DMSC surface (initial state), *E*_CH_3_H_ is the energy of the optimized structure with CH_3_ and H adsorbed on the DMSC surface (final state), *E*_surface_ is the energy of the optimized structure of the DMSC surface with no adsorbate, and *E*_CH_4__ is the energy of the optimized CH_4_ molecule in the gas phase. *E*_a_ is the difference between the energy of the transition state (*E*_TS_) and the energy of the initial state, 
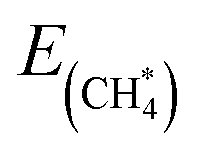
, for the C–H activation step as the system translates from the initial state to the final state.

Transition metals M1 and M2 were selected from the 3d, 4d, and 5d series of the periodic table, yielding a total of 351 unique DMSC surface combinations. Because the M1 and M2 active sites were non-equivalent, two distinct CH_4_ adsorption configurations were considered for every heteronuclear catalyst: (a) 
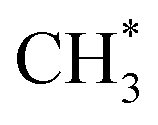
 adsorbed on M1 with H* adsorbed on M2 and (b) H* adsorbed on M1 with 
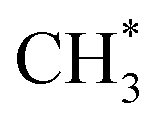
 adsorbed on M2. By accounting for both directional possibilities across the heteronuclear pairs, alongside the homonuclear combinations, a total of 676 distinct methane activation scenarios were rigorously evaluated using DFT. For ML model development, an 80 : 20 train-test split and multiple ML algorithms were employed. This included tree-based algorithms such as random forest regression (RFR), gradient boosting regression (GBR), and extra tree regression (ETR), which leverage the power of an ensemble of decision trees to make predictions. Others are non-parametric algorithms such as support vector regression (SVR) and *k*-nearest neighbour (KNN) regression.

Among the ML models, KNN regression is a non-parametric, distance-based machine learning algorithm that leverages the proximity or similarity of data points to make predictions.^28^ To leverage higher dimensions for better predictions, SVR is a robust algorithm that elegantly and efficiently uses the “kernel trick”. The basic idea is to project the feature space into a higher-dimensional space (potentially an infinite-dimensional space if a radial basis function (RBF) kernel is used), where SVR finds the best-fitting hyperplane for regression. For tree-based algorithms, such as RFR, GBR and ETR, multiple decision trees work in tandem to arrive at the final prediction, which is the average of the predictions of all the decision trees.^29,30^ The nature of these ensemble decision trees can be studied under two primary heads: the manner of tree generation and the manner of decision node splitting. If decision trees are generated in parallel and independently and optimal splitting is performed at each decision node, this ensemble algorithm is termed RFR. Furthermore, GBR is built on the principle of boosting, wherein decision trees are generated sequentially such that each successive tree learns from the errors of its predecessors to progressively improve performance. Specifically, GBR constructs each new tree using the residuals between the predicted and target properties from the previous tree as its input, allowing the model to iteratively correct its own errors. Performing optimal splitting at each decision node, as done in RFR and GBR, is computationally expensive, especially as the size and dimensionality of the dataset increase. ETR addresses this limitation as a faster variant, introducing additional randomization into the construction of the ensemble by selecting decision node splits randomly rather than searching for the optimal split.

The Python package ‘Scikit-learn’^31^ was used to import these algorithms. Moreover, an artificial neural network (ANN) from ‘TensorFlow’^32^ was deployed for model development. ANNs are computationally expensive and data-intensive models mimicking the functioning of the human brain to achieve pattern recognition and capture complex relationships. Multi-layer perceptron (MLP), a type of ANN with 3 or fewer hidden layers, was deployed in this study.

The DFT-obtained data consisted of 
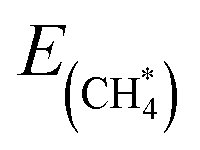
, *E*_a_, and 
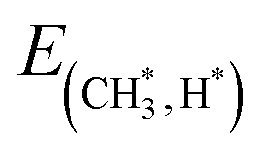
 as the target variables for the ML models. The feature space of the database comprised readily available properties of transition metals, which can be categorised into three classes: d-band-related properties, periodic properties, and previously reported properties. The d-band-related features included the d-band centre, d-band filling, and coupling matrix.

In contrast, the periodic properties included the atomic mass, atomic number, group, period, radius, ionisation energy, melting point, boiling point, heat of fusion, and electronegativity. The atomic polarizability, atomic chemical potential, effective nuclear charge, work function, effective electron density, and atomic hardness are some previously reported properties that formed part of the feature space (Fig. 3). The final step of model performance analysis unraveled the accuracy of all the employed ML algorithms by providing the importance of each feature used. Because the selection of optimized hyperparameters is critical for robust model training, a comprehensive range of hyperparameter values was considered (Table S1) and tested *via* Grid Search in Scikit-learn. Subsequently, ML model performance assessments were conducted using the root mean squared error (RMSE) criterion in this study. To eliminate bias in the database, the models were trained and tested using 100 random train-test splits coupled with 5-fold cross-validation. Ultimately, an averaged RMSE value over these 100 independent trials was reported.

## Results and discussion

**Fig. 4:**
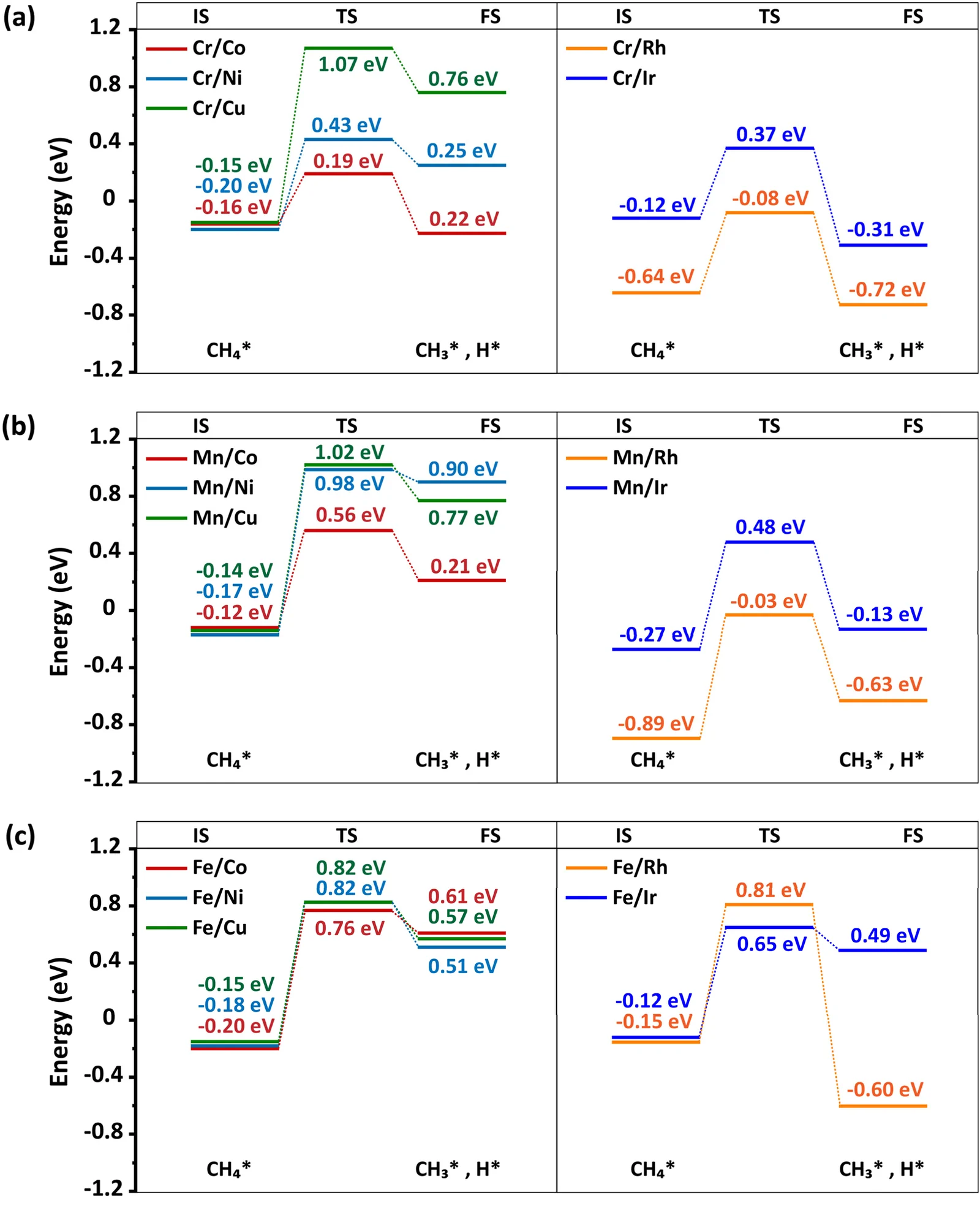
C–H bond activation energy trend for methane on DMSCs constructed using M1 transition metals: (a) Cr, (b) Mn, and (c) Fe, combined with M2 transition metals belonging to the same row (Co, Ni, and Cu) and the same group (Co, Rh, and Ir) in the periodic table. Results for other combinations of transition metals are provided in the SI.

A representation of the active centres in the DMSC is shown in Fig. 2, wherein their positions are marked as M1 and M2. Methane activation on these DMSC active sites was simulated using DFT calculations, where a combination of M1 transition metals (Cr, Mn, and Fe) was paired with M2 metals along the same period (Co, Ni and Cu from the 3d series) and along the same group (Co, Rh and Ir from group 9) of the periodic table. The DMSC combinations in which M1 is Cr and M2 is 3d metals (Co, Ni, Cu) are hereby referred to as Cr/3d combinations, and consequently, Mn/3d and Fe/3d are designated for Mn- and Fe-based DMSCs. A similar nomenclature is followed for the DMSC combinations with M1 as Cr and M2 as group 9 metals (Co, Rh, or Ir), denoted as Cr/G9, and similarly, the labels Mn/G9 and Fe/G9 are used. These subsets were chosen because they represent DMSC pairs for which fully converged, structurally consistent CH_4_ activation pathways (IS, TS, and FS) were obtained across the entire span of both the period (3d series) and group (group 9). The reaction diagrams for the C–H activation step on these DMSCs are represented in Fig. 4.

The DFT calculations (Fig. 4) reveal that replacing the M2 atom progressively tunes the *E*_a_, though this electronic interplay is highly dependent on the identity of the primary M1 site. For early transition metals (Cr and Mn), increasing the d-state filling of the M2 atom significantly hinders C–H activation. The *E*_a_ values show a clear upward trajectory, Cr/Co (0.35 eV) < Cr/Ni (0.63 eV) < Cr/Cu (1.22 eV), mirroring the trend in the Mn/3d series (0.68 eV < 1.15 eV < 1.16 eV). Conversely, substituting M1 with Fe nullifies this electronic sensitivity. Across the Fe/3d series, the activation barriers plateau, Fe/Co (0.96 eV), Fe/Ni (1.00 eV), and Fe/Cu (0.97 eV), suggesting that the Fe active site dominates the catalytic interaction, rendering the role of the adjacent M2 metal insignificant during the C–H bond cleavage step.

**Fig. 5:**
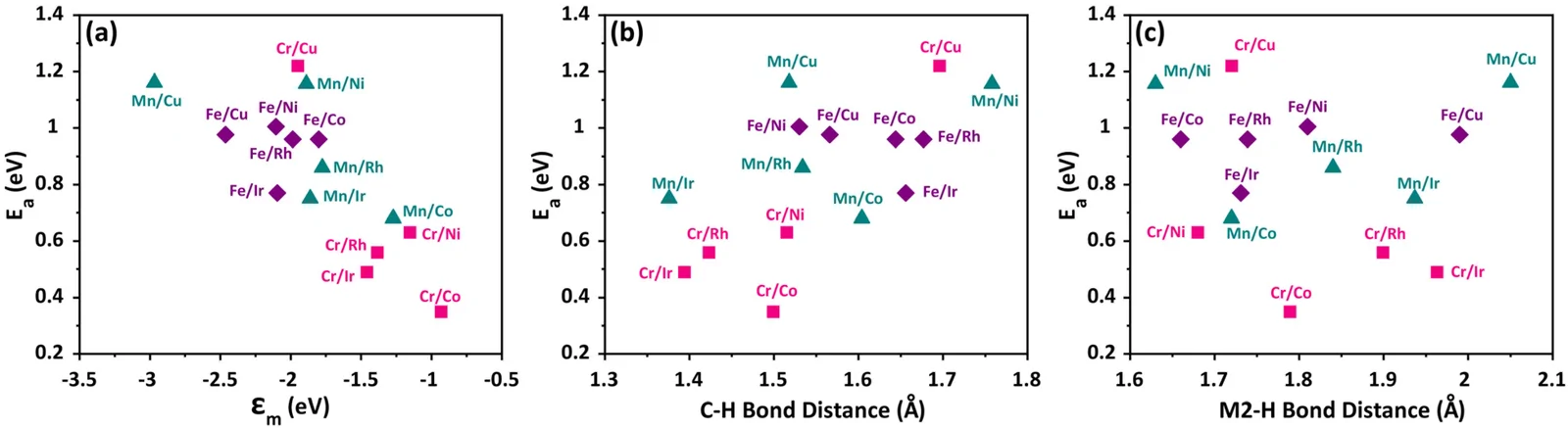
Activation energy (*E*_a_) as a function of the (a) average d-band centre (*ε*_m_), (b) C–H bond distances in the TS structure and (c) M2–H bond distances in the TS structure for the DMSC configurations shown in Fig. 4. All the bond distances, M1–C, M1–H, M2–H, M1–M2 and C–H (C and H from CH_4_), as shown in Fig. 2, are reported in Fig. S1.

To translate these activity trends into structural insights, the *E*_a_ was correlated with the critical C–H bond elongation and the associated M2–H bond distances (Fig. 5(b) and (c), respectively). For instance, in the Cr/3d series, the C–H bond distance in the transition state increases monotonically as the kinetic barrier increases: Cr/Co (1.50 Å) < Cr/Ni (1.52 Å) < Cr/Cu (1.70 Å). This reveals that the highly active Cr/Co catalyst facilitates an “early” transition state, requiring minimal C–H bond stretching to achieve cleavage. Conversely, the less-active Cr/Cu dual-site demands a highly distorted, “late” transition state.

For the Mn/3d series, the C–H distance follows the order Mn/Co (1.60 Å) < Mn/Ni (1.80 Å) > Mn/Cu (1.50 Å), which does not strictly follow the activation-barrier trend Mn/Co (0.68 eV) < Mn/Ni (1.15 eV) ≈ Mn/Cu (1.16 eV). Notably, the barriers for Mn/Ni and Mn/Cu differ by only 0.01 eV, which effectively degenerates within the precision of the presented DFT framework; hence, the comparatively larger C–H bond distance calculated for Mn/Ni does not contradict the early/late TS picture established for the Cr/3d series.

The Fe/3d series shows a similarly compressed energy landscape, with all three barriers falling within a similar range, Fe/Co (0.96 eV), Fe/Ni (1.00 eV), and Fe/Cu (0.97 eV). The corresponding C–H distances, Fe/Co (1.60 Å) > Fe/Ni (1.53 Å) < Fe/Cu (1.57 Å), show no strict monotonic trend. This suggests that the relationship between the activation barrier and the C–H bond distance is a descriptor of the TS character when the activation barriers span a wide energetic range (as is the case for Cr/3d, 0.35–1.22 eV), but this relationship weakens when the barriers are close in value. In such cases, the transition-state geometry is no longer controlled by a single descriptor, but instead reflects a combination of electronic changes.

Moreover, descriptors like M2–H bond distance provide additional insight into Cr/3d, Mn/3d and Fe/3d. Initially, no regular trend emerges for M2–H distances in either the Cr/3d (Cr/Co (1.80 Å) > Cr/Ni (1.68 Å) < Cr/Cu (1.72 Å)) or Mn/3d (Mn/Co (1.70 Å) > Mn/Ni (1.60 Å) < Mn/Cu (2.00 Å)) series. By contrast, the Fe/3d series uniquely shows a clean, monotonically increasing M2–H distance (Fe/Co (1.70 Å) < Fe/Ni (1.80 Å) < Fe/Cu (2.00 Å)), indicating the progressive weakening of the M2–H interaction from Co to Cu. This indicates that the identity of the M1 metal plays a vital role in tuning the overall activation barrier.

For additional analysis, d-band calculations (Fig. S3 and S5) were performed for M1 and M2, as the d-band characteristics of a transition metal can serve as a descriptor of its reactivity. Herein, a “mean d-band centre (*ε*_m_)” was calculated to account for the synergistic electronic effect of both the transition metals. It was defined as the average of the d-band centres of M1 and M2 in the aforementioned combinations of the DMSCs. As shown in Fig. 5(a), in some cases of DMSCs, an inverse relationship between *E*_a_ and *ε*_m_ can be traced, showing that a larger distance of the average d-band centre below the Fermi level relates to a higher activation barrier for C–H bond breakage. For instance, a steady *E*_a_ increment is seen as *ε*_m_ shifts away from the Fermi level for the Cr/3d and Mn/3d DMSCs, denoted as M1/M2 (*ε*_m_), in the order of Cr/Co (−0.9 eV) < Cr/Ni (−1.2 eV) < Cr/Cu (−2 eV) and Mn/Co (−1.3 eV) < Mn/Ni (−1.9 eV) < Mn/Cu (−3 eV). However, this is not generally true, and there are clear outliers from this descriptor, such as Fe/3d combinations, where *E*_a_ and *ε*_m_ do not directly correlate because *E*_a_ (Fe/Co (0.96 eV) ≈ Fe/Ni (1 eV) ≈ Fe/Cu (0.97 eV)) is nearly the same, while *ε*_m_ (Fe/Co (−1.8 eV) < Fe/Ni (−2.1 eV) < Fe/Cu (−2.5 eV)) apparently shifts away from the Fermi level with increased filling of the d-states. A linear fit of *E*_a_*vs. ε*_m_ outputs the *R*^2^ values of 0.987, 0.623 and 0.03 for Cr/3d, Mn/3d and Fe/3d, respectively. Thus, *ε*_m_ may serve as a satisfactory qualitative descriptor for widely distributed *E*_a_ values, such as for Cr/3d combinations. On the contrary, when *E*_a_ values are concentrated, such as in the cases of Mn/Ni (1.15 eV) and Mn/Cu (1.16 eV) as well as Fe/Co (0.96 eV), Fe/Cu (0.97 eV), and Fe/Ni (1 eV), *ε*_m_ fails to accurately capture the quantitative trend. This is evident from the relatively lower *R*^2^ values of 0.623 and 0.03 for the respective cases.

Furthermore, a similar exploration of DMSCs was conducted by keeping the M1 transition metal as Cr, Mn, and Fe while varying M2 across group 9, *i.e.*, Co, Rh, and Ir (Fig. 4). An increase followed by a dip in the *E*_a_ is displayed by the Cr/G9 and Mn/G9, such that Cr/Co (0.35 eV) < Cr/Rh (0.56 eV) > Cr/Ir (0.49 eV) and Mn/Co (0.68 eV) < Mn/Rh (0.86 eV) > Mn/Ir (0.75 eV) as the M2 metal is varied across group 9 transition metals, suggesting that the catalytic activity does not vary uniformly upon descending the group. In contrast, for Fe/G9 DMSCs, there is an effective downward trend, Fe/Co (0.96 eV) ≈ Fe/Rh (0.96 eV) > Fe/Ir (0.77 eV), indicating that the choice of M1 influences how the activation barrier responds to the change in M2 across the group.

The bond distances calculated for the TS structure are reported in Fig. S1. The M2–H bond distances are observed to follow an effective upward trend for Cr/G9, Mn/G9 and Fe/G9 DMSC combinations in the orders of Cr/Co (1.7 Å) < Cr/Rh (1.9 Å) < Cr/Ir (1.96 Å), Mn/Co (1.7 Å) < Mn/Rh (1.8 Å) < Mn/Ir (1.9 Å), and Fe/Co (1.66 Å) < Fe/Rh (1.74 Å) ≈ Fe/Ir (1.73 Å), respectively, suggesting a progressively weaker M2–H interaction as M2 descends the group. Examining the C–H bond distance in the TS structure reveals the declining trends Cr/Co (1.5 Å) > Cr/Rh (1.42 Å) > Cr/Ir (1.39 Å) and Mn/Co (1.6 Å) > Mn/Rh (1.5 Å) > Mn/Ir (1.4 Å) and the nearly unchanging trend Fe/Co (1.64 Å) ≈ Fe/Rh (1.68 Å) ≈ Fe/Ir (1.66 Å). Because the C–H and M2–H distances move in opposite directions across the group, a consistent correlation between *E*_a_ and the bond distances is not evident. Moreover, d-band calculations were performed for M1 and M2, and DOS representations are described in Fig. S5. As *E*_a_ decreases from Fe/Co (0.96 eV) to Fe/Ir (0.77 eV), the *ε*_m_ moves away from the Fermi level from Fe/Co (−1.8 eV) to Fe/Ir (−2.1 eV), consistent with the expected inverse relationship between *ε*_m_ and catalytic activity. However, a mismatch is observed for Cr/G9 and Mn/G9 DMSCs, where the *ε*_m_ shifts consistently away from the Fermi level from Cr/Co (−0.9 eV) to Cr/Ir (−1.5 eV) and from Mn/Co (−1.3 eV) to Mn/Ir (−1.9 eV), while *E*_a_ initially increases, followed by a dip for both series, indicating that *ε*_m_ alone is insufficient to fully capture the barrier trend in these cases.

Overall, these results indicate that besides the critical geometric features like C–H and M2–H bond distances, which are vital to C–H bond activation, even electronic features such as *ε*_m_ are unable to capture the variations in *E*_a_ across different DMSC configurations. The lack of a universal trend in *E*_a_ with traditional descriptors, such as periodic, d-band, and geometric properties, underscores the need for alternative approaches to identify and understand these trends. Machine learning offers a promising avenue in this regard.

By leveraging data-driven models and machine learning techniques, it is possible to uncover complex relationships and patterns in the data that may not be evident from traditional descriptors alone. Machine learning algorithms can be trained on a dataset of known activation energies and other relevant features to develop predictive models, enabling the prediction of *E*_a_ or other descriptors for new catalyst systems. This approach provides an opportunity to gain insights into the factors influencing reactivity and guide catalyst design in a more efficient and targeted manner. To implement this, a comprehensive database was generated by accounting for different DMSC configurations by querying M1 and M2 across the entire transition metal block shown in Fig. 2, and C–H activation was performed on those configurations using DFT.

**Fig. 6:**
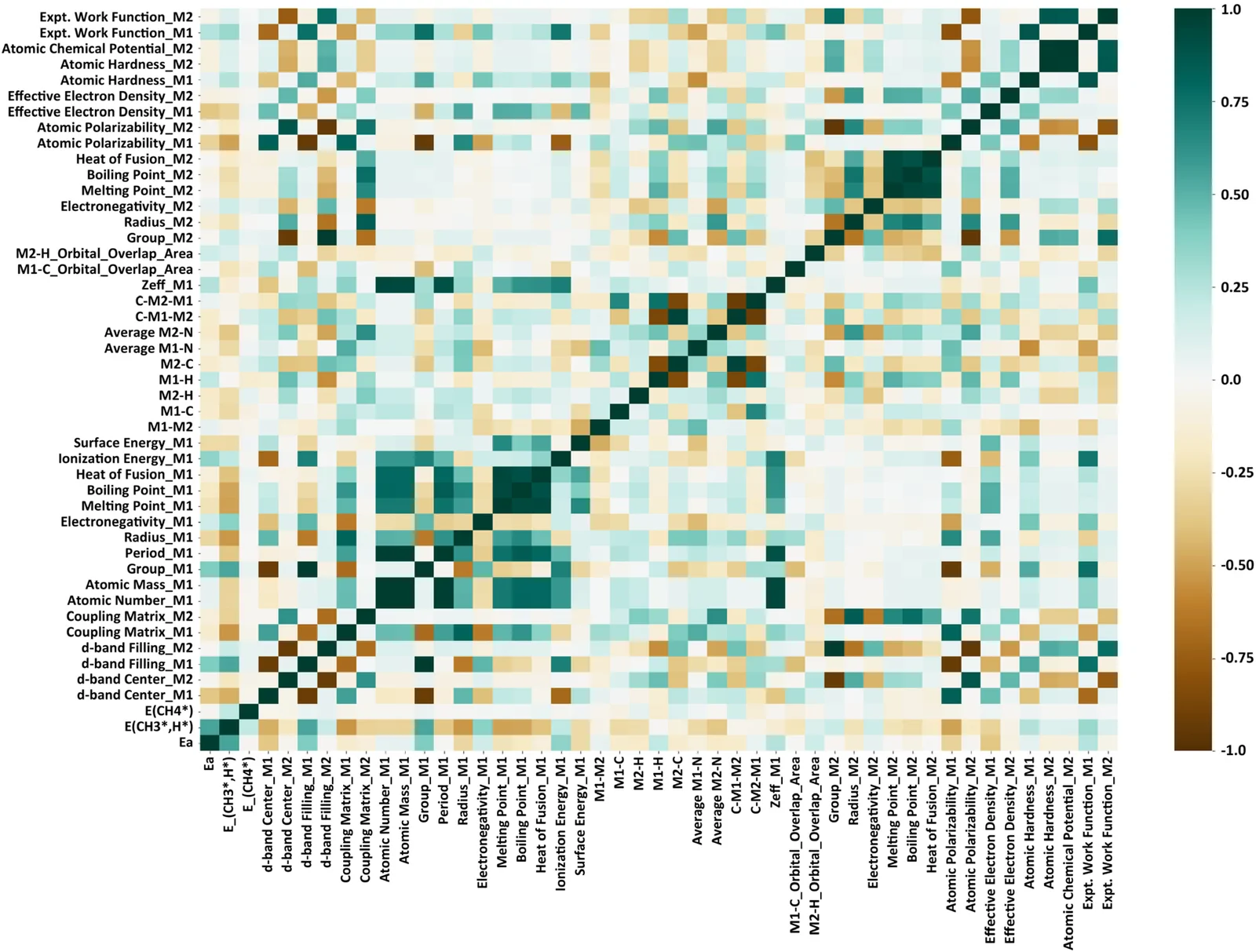
Pearson correlation plot depicting linear dependency between the feature space and target space.

The ML workflow shown in Fig. 3 is described in the Methodology section, from data acquisition through model testing and performance analysis. A Pearson correlation plot (Fig. 6) is also shown to provide a visual representation of the nature of the relationships between the target variables *E*_a_, 
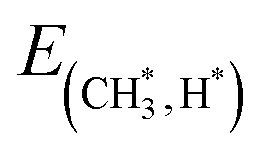
 and 
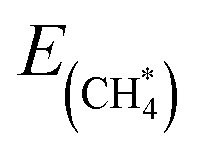
 and the feature space. Feature pairs exhibiting substantial collinearity (|*r*| > 0.8) were identified, and for each such pair, the feature showing a weaker correlation with the target property (*E*_a_ and 
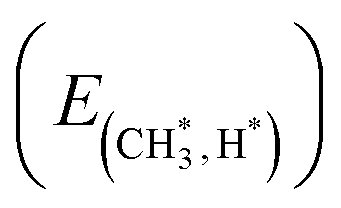
) was removed (Fig. S9). This systematic screening reduced the feature set from 42 descriptors to 27 descriptors for *E*_a_ (Fig. S10) and 22 descriptors for 
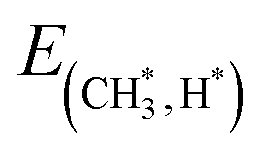
 (Fig. S11), eliminating redundancies such as the perfect correlation (*r* = 1.00) between Group_M1 and d-band filling_M1, which reflects the deterministic relationship between the group number and d-electron count for transition metals.

A higher absolute value of the correlation coefficient indicates a stronger linear relationship between the associated quantities. In contrast, a lower absolute magnitude indicates either the absence of a relationship between those quantities or the existence of a complex, nonlinear relationship. Herein, a brief account of the dataset's nature is provided.

By screening all 676 DMSC combinations for irregularities, such as bond breakage and protrusion of doped atoms after CH_4_ activation, the dataset was narrowed down to 519 combinations. Further, after NEB calculations, 302 transition states were estimated and hence considered for the ML study. An outlier detection technique called the *Z*-score methodology assisted in removing the outliers from the dataset (Fig. S6(a)–(c) show the frequency distributions), and as a result, a final dataset of 237 datapoints with the energetics of the IS, TS and FS structures for methane activation on DMSCs was used in the ML model.

Heteronuclear DMSCs (different metal dopings at dual sites) are observed to dominate the lower range of *E*_a_ for C–H bonds, with 87 candidate DMSCs in the range of 0.10–0.50 eV, compared to only 17 above 1.50 eV. Moreover, as much as 75% of all the heteronuclear DMSCs exhibit *E*_a_ of less than 1 eV. Examples such as Hf/Ir, Tc/V, Au/Mo, Mo/Fe, Ru/Co and Mo/Ni possess the lowest *E*_a_ in the range of 0.10–0.50 eV, while other DMSCs like Ni/Rh, Cu/Mn, Cu/Cr, Ir/V lie above the 1.60 eV mark. With the same metal doping on both sites, homonuclear DMSCs also show some interesting cases, with more than 50% of those combinations having *E*_a_ below 1 eV. Some of the lowest *E*_a_, below 0.50 eV, are observed for Zr/Zr, Mn/Mn, V/V, and Rh/Rh, whereas Pd/Pd, Pt/Pt, and Ir/Ir show *E*_a_ on the higher end, above 1.50 eV.

The final DFT data set (237 data points) was used to train six ML algorithms (*viz.* KNN, SVR, RFR, GBR, ETR, and ANN) over a range of hyperparameters, as listed in Table S1. After tuning the KNN hyperparameter to 5 nearest neighbors, test RMSE values of 0.33 eV and 0.42 eV are obtained for *E*_a_ and 
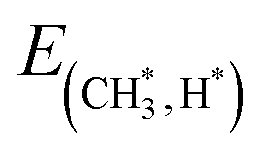
, respectively. Similarly, using the RBF kernel, gamma = 0.02, and *C* = 1000 as tuned hyperparameters, test RMSE values of 0.29 eV and 0.38 eV are obtained for *E*_a_ and 
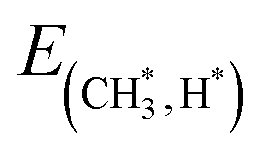
, respectively, for the SVR algorithm. By contrast, ANN predictions show the test RMSE values of 0.32 eV and 0.39 eV for *E*_a_ and 
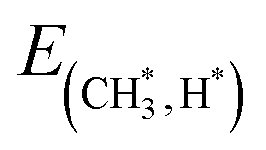
, respectively.

**Fig. 7:**
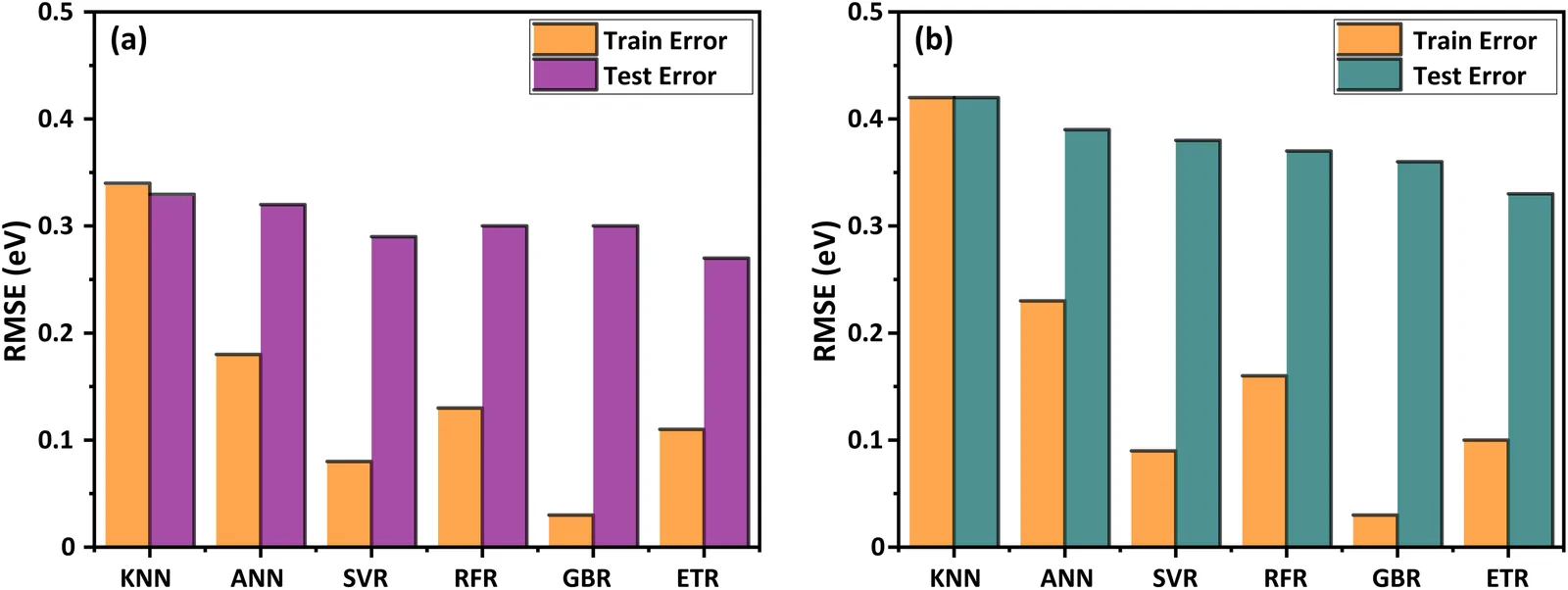
Comparison of ML model performance based on RMSEs for the (a) activation energy (*E*_a_) and (b) final state adsorption energy 
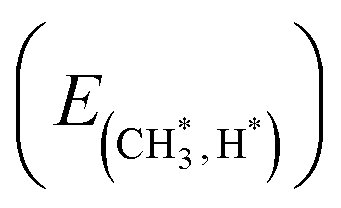
.

Given the complex nature of the C–H activation problem, including high nonlinearity and a considerable feature space, ensemble methods in conjunction with decision trees offer a powerful approach to capture complex trends and relationships. Some algorithms, such as RFR, ETR and GBR, are classified under this category and were explored for further ML predictions. RFR performance in this study is rated at test RMSEs of 0.30 eV and 0.37 eV for the predictions of *E*_a_ and 
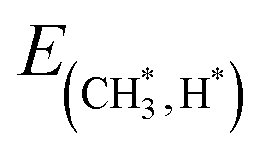
, respectively, with the number of estimators tuned to 300 and the maximum tree depth to 7. Predictions from GBR translated to test RMSEs of about 0.30 eV and 0.36 eV for *E*_a_ and 
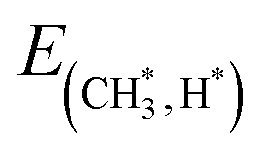
, respectively, which performs marginally better than RFR in this case. Hyperparameter tuning calibrated the number of estimators to 400, the learning rate to 0.03 and the maximum tree depth to 4 for the same. ETR proves to be the best performer among all ML algorithms, with 200 estimators and a maximum tree depth of 7 as tuned hyperparameters. *E*_a_ and 
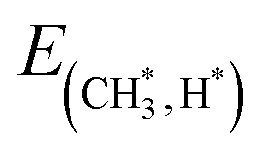
 test RMSE values for the same are 0.27 eV and 0.33 eV, respectively, as shown in Fig. 7. In addition, the calibrated hyperparameters utilized to train the ML models are summarized in Table 1.

**Table 1:** Optimised hyperparameters of ML models for *E*_a_ and 
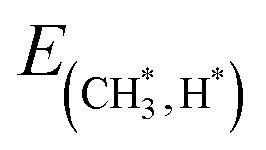
 prediction

Models	Hyperparameter	Values
KNN	Number of nearest neighbors	5
SVR	Kernel	Radial basis function
	Kernel coefficient (gamma)	0.02
	Regularization parameter (C)	1000
RFR	Number of decision trees	300
	Maximum depth of the tree	7
ETR	Number of decision trees	200
	Maximum depth of the tree	7
GBR	Number of decision trees	400
	Learning rate	0.03
	Maximum depth of the tree	4
ANN	Activation function	Hyperbolic tangent
	Loss function	Mean square error
	Optimizer	Stochastic gradient descent

Across the six ML algorithms evaluated, the train and test RMSE values for *E*_a_ (Fig. 7) provide additional insights into how consistently each model performs between the training and test data. ETR achieves the lowest test RMSE (0.27 eV) among the six models and shows a relatively small difference between its train and test RMSEs (0.11 eV and 0.27 eV, respectively), indicating that its additional layer of split-level randomization, beyond RFR's optimal-splitting approach, contributes to more stable and consistent predictions across the dataset. GBR and ANN show comparatively larger differences between train and test RMSEs (0.03 eV to 0.30 eV and 0.18 eV to 0.32 eV, respectively). For ANN, this is consistent with the general tendency of neural networks to require larger datasets to reliably learn complex nonlinear mappings, while GBR's sequential, residual-driven tree construction may make it more sensitive to the size of the current dataset. RFR and SVR show intermediate train-test differences (0.13 eV to 0.30 eV and 0.08 eV to 0.29 eV, respectively), with test performance comparable to, though marginally higher than, that of ETR. KNN is the only model for which the train RMSE (0.34 eV) exceeds the test RMSE (0.33 eV), which reflects the leave-one-out approach applied to its train-error estimate to avoid the artificially low values caused by self-matching in distance-weighted KNN. In summary, ETR is utilized for modelling *E*_a_ in this study, given the lowest test RMSE and the comparatively consistent performance between training and test data.

**Fig. 8:**
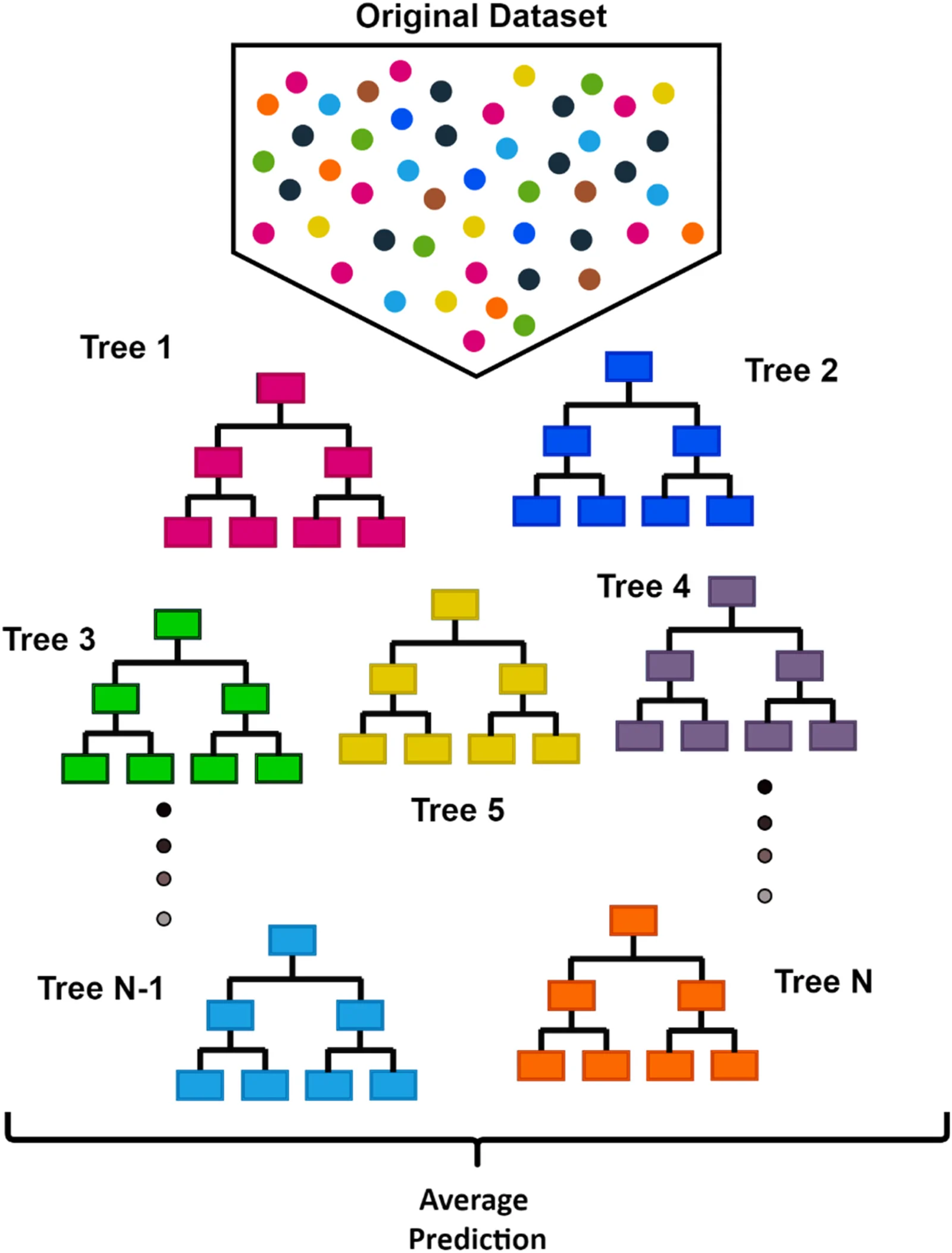
Working of extremely randomised tree regression (ETR), also known as the extra tree regression model.

A pictorial representation of the working of the ETR algorithm is given in Fig. 8. It is a faster variant of RFR, as it introduces randomisation into the construction of the ensemble of decision trees by randomly splitting each decision node rather than using optimal splitting.

**Fig. 9:**
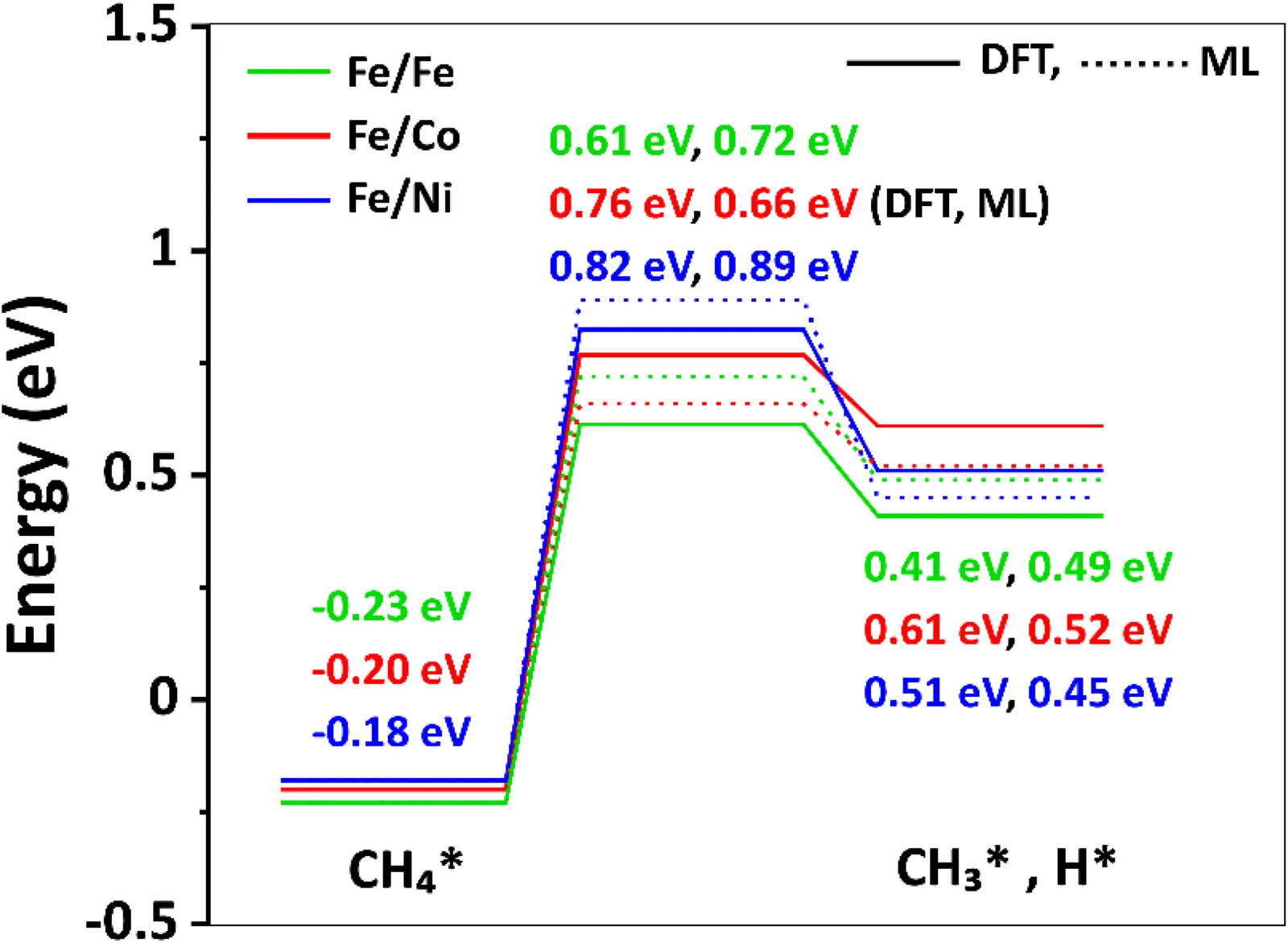
Representation of the DFT-computed (solid) and ML-predicted (dashed) reaction energetics for C–H activation from the best-performing ETR model.

**Fig. 10:**
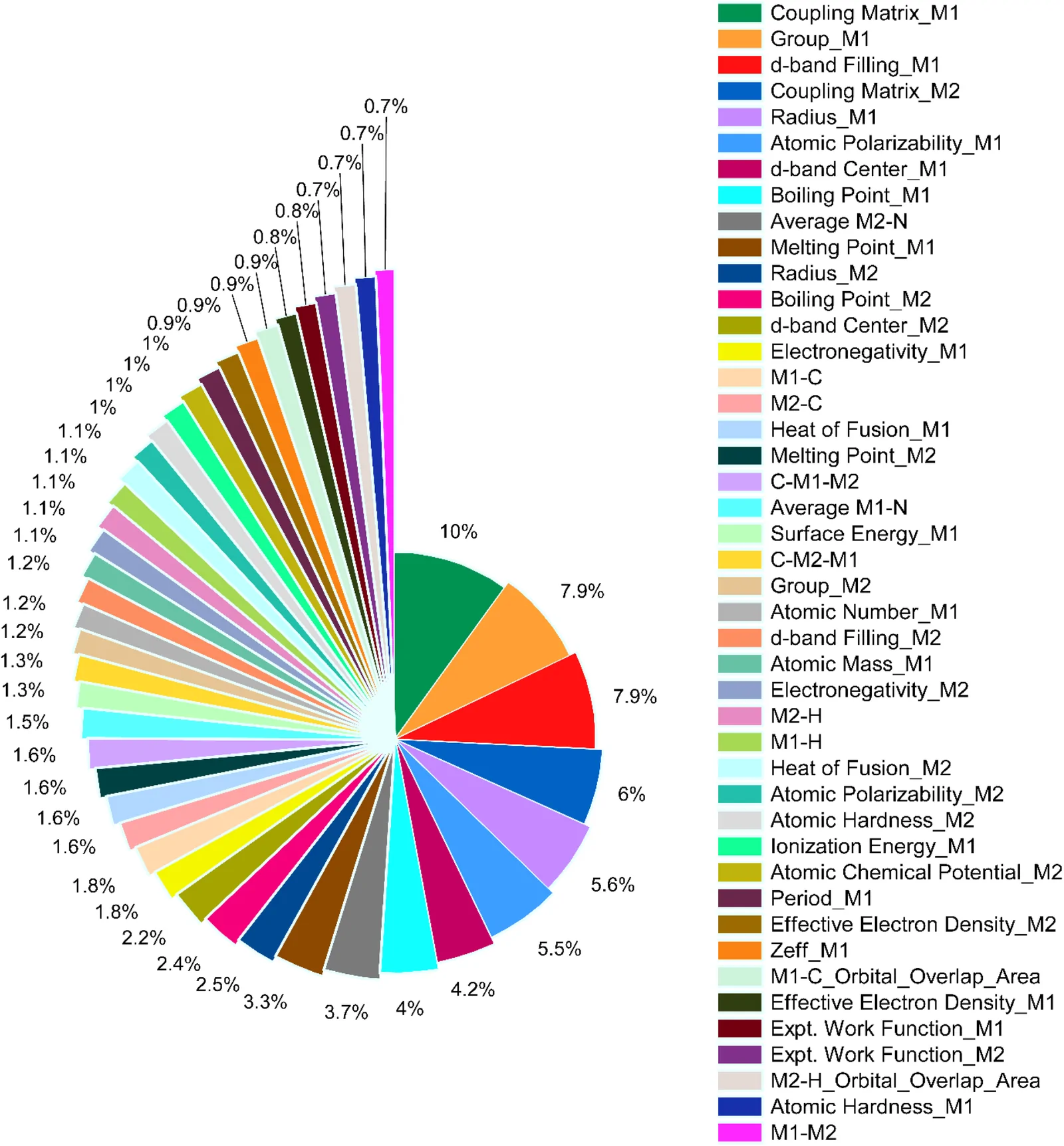
Feature importance for 
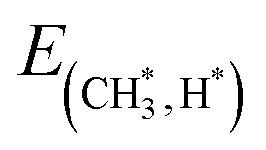
 prediction using the ETR model.

Based on the best-performing ETR model, Fig. 9 shows the ML-predicted and DFT-calculated reaction energy diagrams for DMSC combinations of Fe/Fe, Fe/Co, and Fe/Ni. The feature-wise relevance in the 
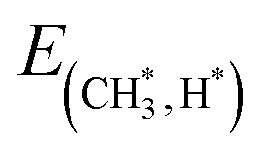
 prediction is depicted in the corresponding feature importance plot in Fig. 10. The coupling matrix of M1 makes the highest contribution (10%) towards 
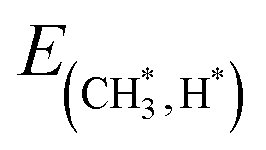
 prediction, followed by other relevant features like the group of M1 (∼8%), d-band filling for M1 (∼8%), coupling matrix of M2 (6%), radius of M1 (>5%) and atomic polarizability of M1 (>5%). The coupling matrix is a crucial property for a transition metal as it directly contributes to the adsorption energy by accounting for the interaction energy between the metal's d electrons and the adsorbate.^33^ We noticed that most of the features with greater importance are those associated with M1, which is the site where CH_4_ activation is proposed to take place, which suggests a strong relationship between the target property and the properties related to M1.

It is evident from the NEB calculations that accurately predicting a transition state is indeed a difficult, computationally expensive, and time-consuming task. Naturally, primary features such as periodic properties (atomic number, atomic mass, ionisation energy, and other properties shown in Fig. 3) and d-band-related properties (d-band centre, d-band filling, and coupling matrix) are expected to be inadequate for predicting *E*_a_. Therefore, some structural features from the DFT calculations were incorporated during *E*_a_ prediction. These include geometric features, such as bond distances and bond angles in the final-state structure, and energy-related features, 
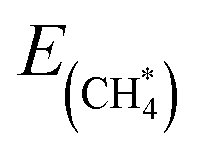
 and 
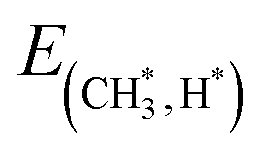
. Given that geometry optimisation is comparatively less computationally expensive than NEB calculations, including these features in the ML study does not significantly alter the efficacy or cost of the ML models in predicting *E*_a_.

**Fig. 11:**
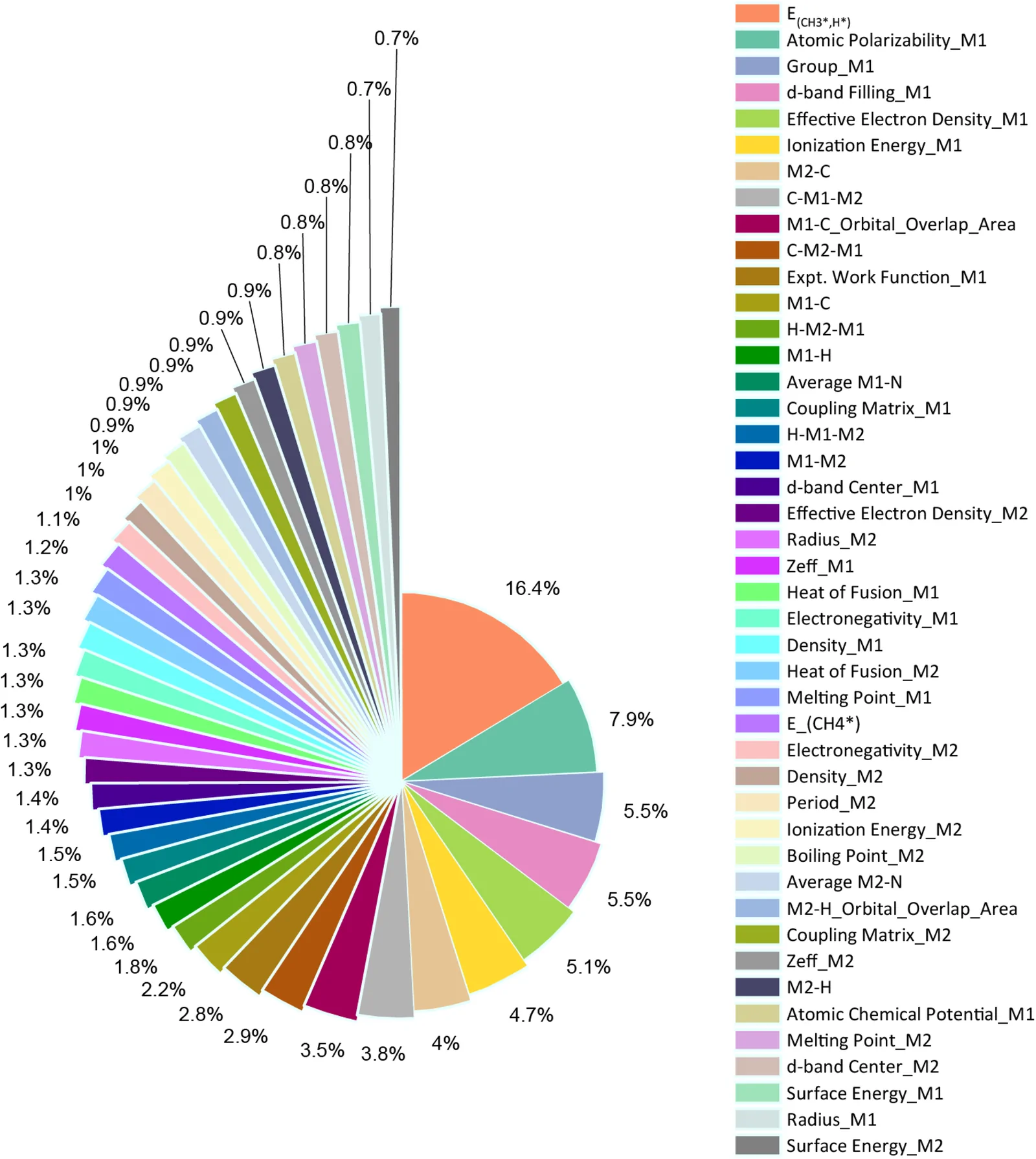
Feature importance for *E*_a_ prediction using the ETR model.

Based on the best-performing ETR model, the feature-wise relevance in the *E*_a_ prediction is depicted in the corresponding feature importance plot in Fig. 11. 
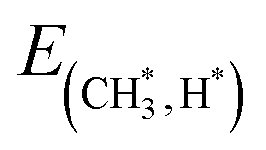
 makes the highest contribution (>16%) towards *E*_a_ prediction, followed by other relevant features like the atomic polarizability of M1 (∼8%), group of M1 (>5%), d-band filling for M1 (>5%) and effective electron density of M1 (>5%). We noticed that most of the features of greater importance are associated with M1, the site where CH_4_ activation takes place; a similar trend is observed in the case of 
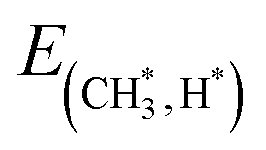
 prediction as well. This suggests an overall correlation between the target properties in this study and the properties of M1. This also shows that 
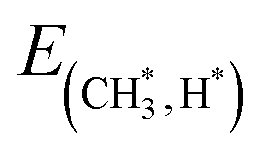
 can be proposed as a significant descriptor for *E*_a_ prediction, suggesting a direction for exploring other potential features that could aid the ML model in accurately predicting *E*_a_, thereby greatly speeding up the transition-state estimation.

The 
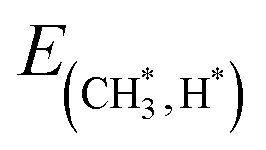
 adsorption energy was identified as the most significant feature for predicting the *E*_a_, with a feature importance of 16.4%. Subsequently, a Brønsted–Evans–Polanyi (BEP) analysis was conducted to evaluate the linear dependency between 
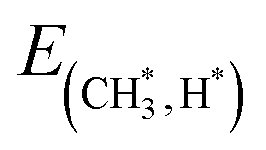
 and *E*_a_, yielding a poor linear fit (*R*^2^ = 0.18), which confirms that these DMSCs bypass traditional linear scaling relationships, as illustrated in Fig. S12. This breakdown is directly driven by synergistic interactions at the dual-metal active sites, which is consistent with previously reported studies on escaping BEP relationships for single-atom alloys and dual-atom catalysts by Darby *et al.*^34^ and Li *et al.*^35^

**Fig. 12:**
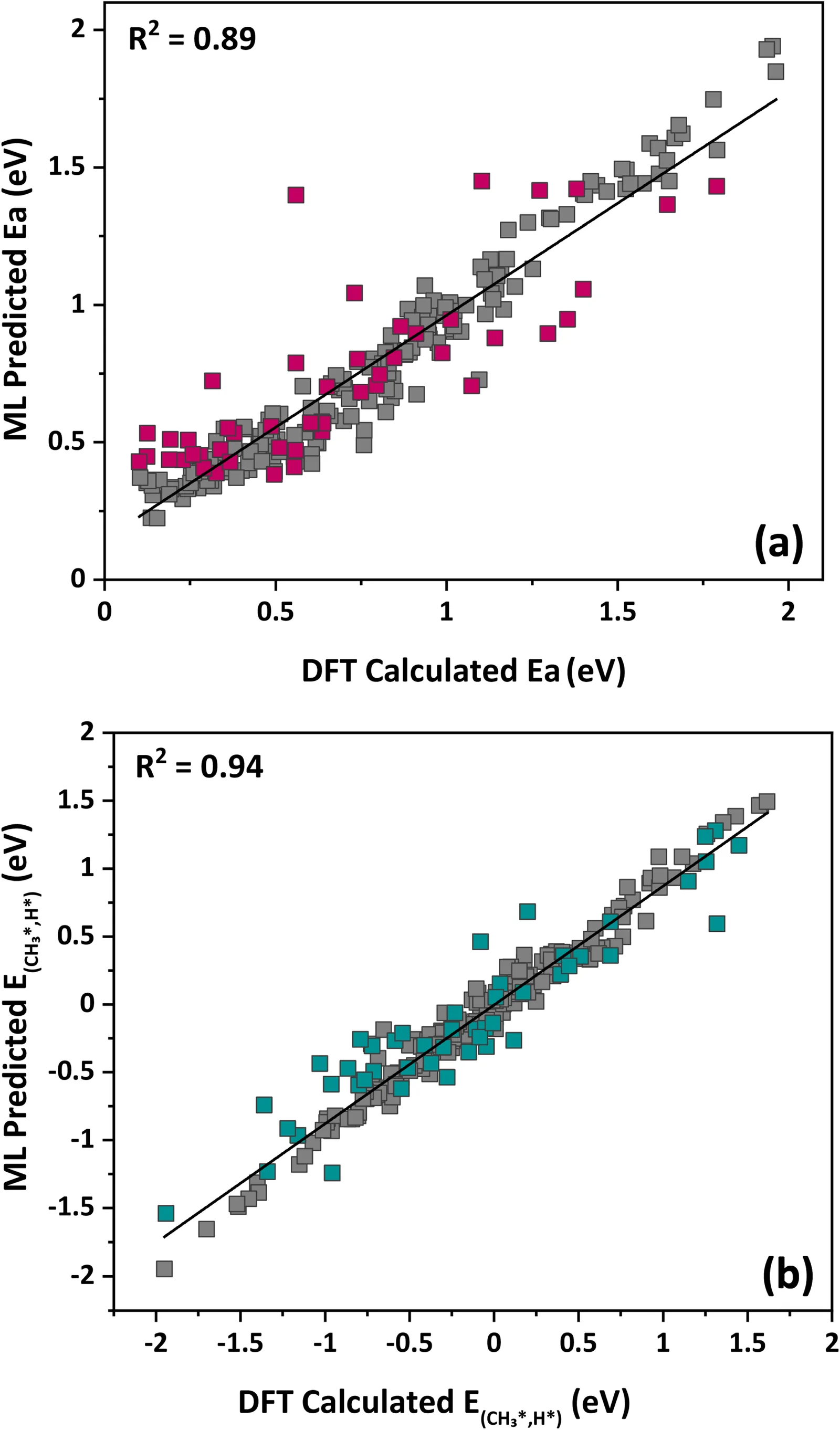
Deviation of the ML-predicted (ETR model) (a) *E*_a_ and (b) 
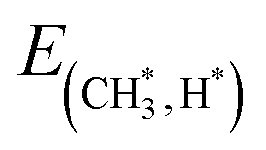
 from DFT calculations.

The accuracy of the best-performing ETR model for predicting *E*_a_ and 
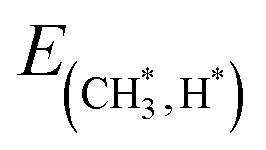
 is shown in Fig. 12, with *R*^2^ values of 0.89 and 0.94, respectively. This suggests that in this study, ML models trained on 80% of the dataset are competent at predicting 20% of the unknown target variables with significant accuracy. Therefore, a combined ML-DFT approach can be a promising route to rapidly screen potential catalysts by quickly estimating transition-state energies, which has been a computationally expensive and time-consuming task in computational chemistry.

Following the successful identification of dual transition-metal sites supported on graphene with low activation barriers for C–H activation, we aimed to further explore their effectiveness and potential as catalysts for the C–C coupling reaction, which leads to the formation of the first C_2_ intermediate, ethane (C_2_H_6_). Among the studied combinations, as shown in Fig. 4, Cr/Co exhibits the lowest C–H activation barrier of 0.35 eV, followed by Cr/Ir (0.49 eV) and Mn/Co (0.68 eV). Other combinations, such as Cr/Ni, Cr/Rh and Fe/Fe, show moderate activation barriers of 0.63 eV, 0.72 eV, and 0.84 eV, respectively. However, Fe/Cu and Fe/Co are comparable, with barriers of 0.97 eV and 0.96 eV, respectively. Systems such as Cr/Cu and Fe/Ni exhibit the highest barriers, measured at 1.22 eV and 1.00 eV, respectively. Although the activation barriers of Fe-based systems are higher than those of Cr-based systems, all Fe-containing catalysts exhibit nearly identical activation barriers, suggesting consistent catalytic behavior across the Fe/3d series. Because Fe is one of the most extensively studied transition metals for methane activation and conversion reactions,^2,10,35–37^ Fe and Cr-based systems were further analysed to understand their behavior in facilitating the C–C coupling reaction.

**Fig. 13:**
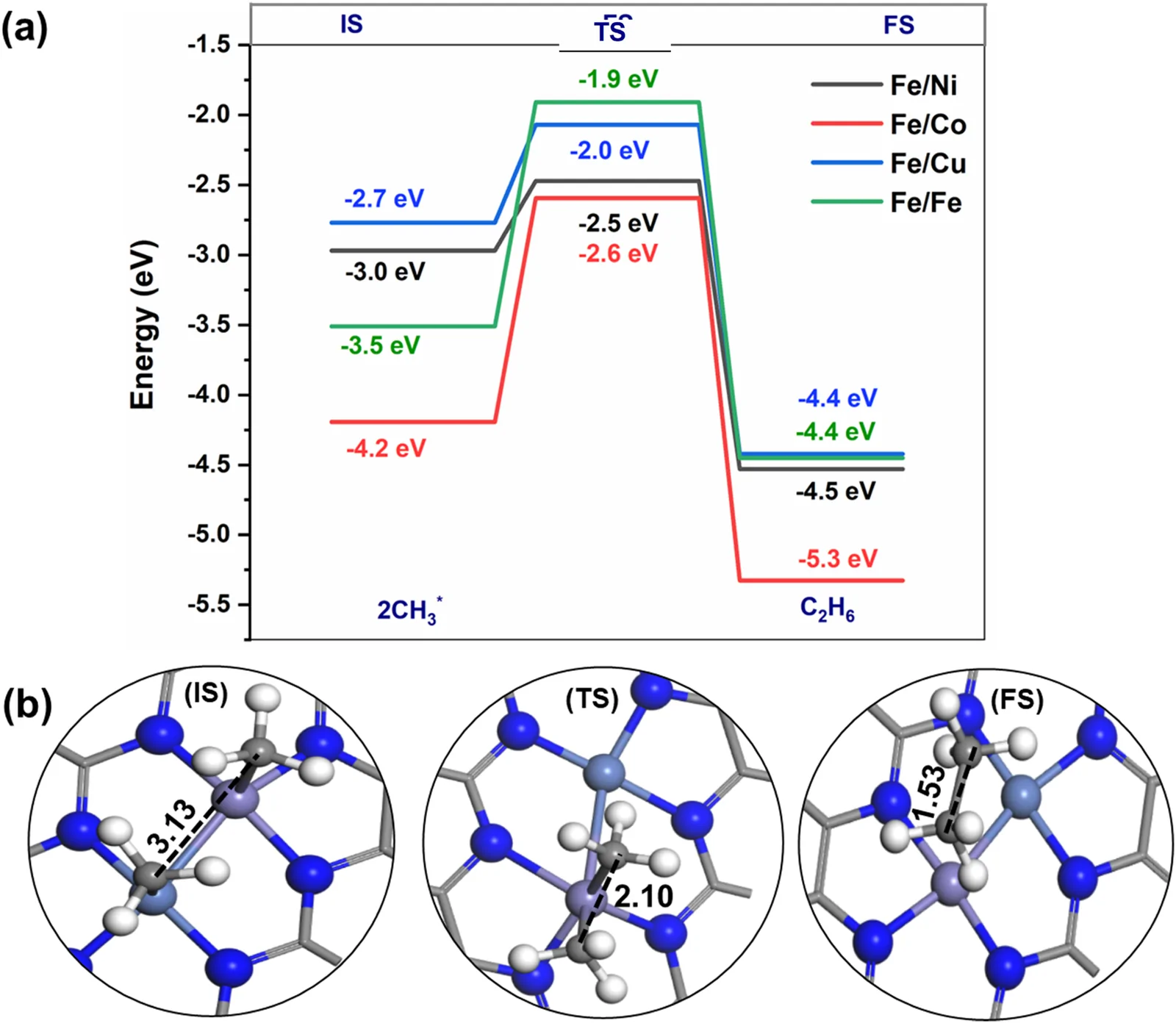
Coupling of two methyl groups to generate ethane. (a) Energy profile illustrating the C–C coupling reaction for Fe-based systems paired with M2 transition metals (Ni, Co, and Cu) and (b) structural details (IS, TS and FS) of Fe/Ni as a representative example.

Focusing initially on the Fe-based systems paired with M2 transition metals (Co, Ni and Cu) belonging to the same row in the periodic table, the coupling of two CH_3_ groups proceeds as illustrated in Fig. 13(b). The energy profiles for Fe/Ni, Fe/Co, Fe/Cu and Fe/Fe are depicted in Fig. 13(a). For Fe/Ni, the initial state (IS) involves two methyl groups absorbed separately on Fe and Ni, with Fe–C and Ni–C bond distances of 1.98 Å and a C–C interatomic distance of 3.13 Å (Fig. 13(b), IS). As the reaction proceeds, the C–C bond distance decreases to 2.10 Å in the TS and further to 1.53 Å in the FS (Fig. 13(b), TS and FS). In the TS, both CH_3_ are attached to Fe at a distance of 2.1 Å. In the FS, ethane desorbs, with the Fe–C and Ni–C bond distances increasing to 3.8 Å and 3.4 Å, respectively.

The *E*_a_ for the C–C coupling step differs significantly among the systems. Fe/Ni exhibits the lowest activation barrier (0.5 eV), followed by Fe/Cu (0.7 eV). In contrast, Fe/Co and Fe/Fe show a higher barrier of 1.6 eV. The reaction energy of the Fe/Ni system, which exhibits the lowest activation barrier for C–C coupling, is calculated to be −1.56 eV, indicating that this step is highly exothermic. Thermodynamically, the coupling reaction is favourable across the Fe-based systems studied, with enthalpy changes (Δ*H*) ranging from −1.65 eV (Fe/Cu) to −0.93 eV (Fe/Fe).

**Fig. 14:**
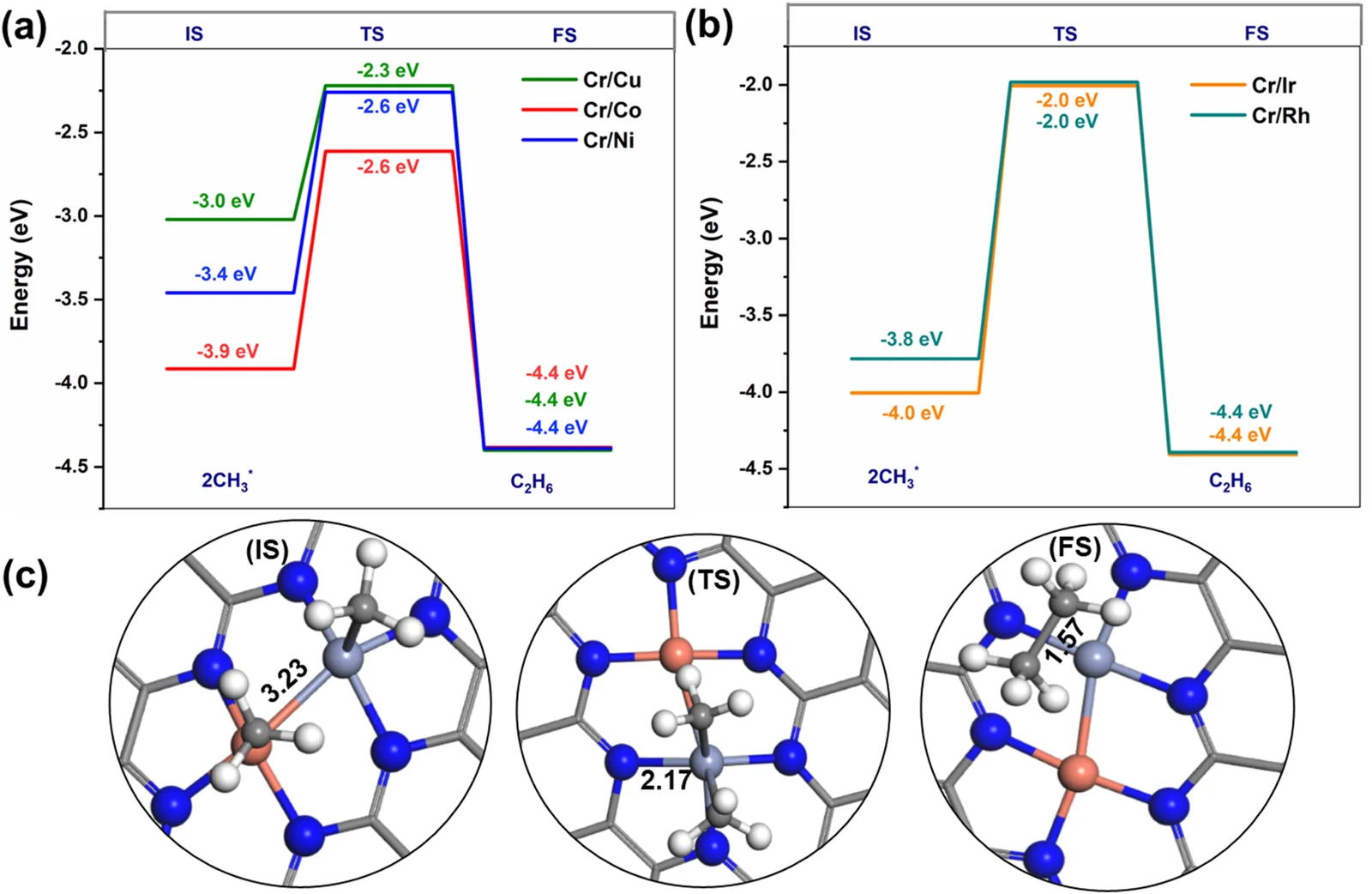
(a and b) Energy profiles depicting the C–C coupling reaction for Cr-based systems paired with M2 transition metals (Ni, Co, Cu, Ir and Rh). (c) Structural insights (IS, TS and FS) for Cr/Cu.

The energy profile of the Cr-based system paired with M2 transition metals (Cu, Co, Ni, Ir, and Rh) is presented in Fig. 14(a) and (b). The IS, TS, and FS structures on Cr/Cu are shown in Fig. 14(c). The IS shows distinct energies, with Cr/Ir being the most stable (−4.0 eV), followed by Cr/Rh (−3.8 eV), Cr/Co (−3.9 eV), Cr/Ni (−3.4 eV) and Cr/Cu (−3.0 eV).

For Cr/Cu in the IS, two methyl groups are adsorbed separately on Cr and Cu, with a C–C distance of 3.23 Å. In the TS, the C–C bond distance decreases to 2.17 Å, with both CH_3_ groups remaining attached to Cr (Fig. 14(c)). In the FS, ethane forms with a C–C bond distance of 1.57 Å, and the Cr–C and Cu–C distances increase as the molecule desorbs from the surface. Among these, Cr/Cu demonstrates the lowest activation barrier of 0.8 eV for C–C coupling, while Cr/Co and Cr/Ni exhibit higher barriers of 1.3 eV and 1.2 eV, respectively. Cr/Ir and Cr/Rh show even higher barriers of 2.0 and 1.8 eV, respectively. The FS energies for all Cr-based systems range from −1.38 (Cr/Cu) to −0.39 eV (Cr/Ir).

All combinations exhibit a similar pattern for C–C coupling: the C–C bond forms, and ethane desorbs. However, the Cr/Co system shows a unique pathway. The coupling reaction involves simultaneous coupling and dehydrogenation of one CH_3_ group, leading to the formation of an intermediate CH_3_CH_2_ species adsorbed on the Cr atom, with the H atom occupying the bridge site between the dual-metal atoms.

Detailed structural insights for Cr/Co (IS, TS, and FS) are provided in Fig. S8. Subsequently, the H atom transfers to the CH_3_CH_2_ intermediate, forming ethane. This step involves a low activation barrier of 0.3 eV. Similar transition states (TS) involving the simultaneous formation of a C–C bond and the dissociation of a C–H bond have also been observed for the Co-based system, as reported by Huang *et al.*^10^ Finally, the Mn-based system shows an activation barrier of 1.6 eV and a reaction energy change of −0.56 eV, as shown in Fig. S7.

To further evaluate the selectivity of the most promising catalysts, additional CI-NEB calculations were performed for the competing CH_3_ dehydrogenation pathway 
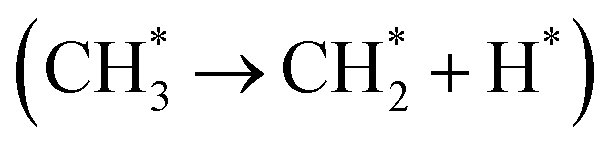
. A similar competition between CH_3_ dehydrogenation and CH_3_–CH_3_ coupling has also been reported by Huang *et al.*,^10^ who showed that Co_2_–N–C exhibits high selectivity toward ethane and greater resistance to deep methane dehydrogenation. Consistent with this observation, the calculated activation barriers for Fe/Cu (2.50 eV), Cr/Ni (1.77 eV), and Fe/Ni (1.65 eV) are consistently higher than the corresponding CH_3_–CH_3_ coupling barriers (0.70, 1.20, and 0.50 eV, respectively). These results indicate that C–C coupling is kinetically favoured over further CH_3_ dehydrogenation on these representative catalysts, supporting the mechanistic conclusion that the selected dual-metal-site catalysts preferentially promote C_2_ formation. The corresponding energy profiles and numerical comparisons are provided in Fig. S14 and Table S5 of the SI. Overall, these findings highlight the potential of Cr- and Fe-based dual-metal-site catalysts to promote C–C coupling while suppressing further dehydrogenation of the CH_3_ intermediate, thereby favoring C_2_ hydrocarbon formation.

## Conclusion

This study provides a comprehensive understanding of C–H bond activation on graphene-supported DMSCs for NOCM through a combined DFT-ML approach. The relationship between the activation energy (*E*_a_) and critical geometric properties, such as C–H and M2–H bond distances, or electronic properties, like average d-band centre (*ε*_m_), shows highly nonlinear trends. To address this complexity, a data-driven approach using ML models is employed to extract and understand these trends. The ETR model emerges as the best performer, accurately predicting *E*_a_ and 
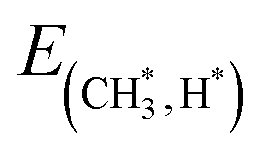
, with RMSEs of 0.27 eV and 0.33 eV, respectively. Through supervised learning, the ML models provide interpretability *via* feature importance analysis, revealing that d-band-related properties and periodic properties play crucial roles in predicting 
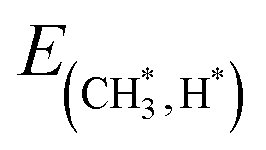
. In addition, the study investigates the C–C coupling mechanism for ethane formation, highlighting the influence of dual-metal-site combinations on the coupling activation barriers. Fe/Ni and Fe/Cu exhibit the lowest coupling activation barriers of 0.5 eV and 0.7 eV, with highly exothermic reaction energies of −1.56 eV and 1.65 eV, respectively, making them the most favourable systems thermodynamically. Overall, this DFT-ML study effectively bridges electronic structure insights with data-driven predictions, facilitating high-throughput screening of potential catalysts and thereby enabling accelerated catalyst discovery for efficient methane activation and conversion.

## Conflicts of interest

There are no conflicts to declare.

## Supplementary Material

RA-OLF-D6RA03770D-s001

## Data Availability

The data supporting this article are included in the main manuscript and supplementary information (SI). Supplementary information is available. See DOI: https://doi.org/10.1039/d6ra03770d.
