# Supplementary material for: A machine learning approach to predict C–H bond activation on dual-site doped graphene catalysts
Source: RSC Adv. 2026 Sep 21;16(44):55844–58. doi: 10.1039/d6ra03770d (PMC13591082; doi:10.1039/d6ra03770d)
Supplement: Supplementary file 1 [file RA-016-D6RA03770D-s001.pdf]

Supplementary Information (SI) for RSC Advances.
This journal is © The Royal Society of Chemistry 2026

A Machine Learning Approach to Predict C-H Bond Activation on Dual-Site Doped Graphene Catalyst

Amrish Kumar [a], Iqra Ahangar [a,b], Shantanu Suryakant Sontakke [a], Manojkumar Ramteke [c], Tuhin S. Khan[d]*, M. Ali Haider*[a,e]

a Renewable Energy and Chemicals Lab, Department of Chemical Engineering, Indian Institute of Technology Delhi, Hauz Khas, New Delhi, 110016, India

b Engineering Technology and Science Division, Department of Chemical Engineering, Higher College of Technology, Abu Dhabi 25026, UAE

c Department of Chemical Engineering, Indian Institute of Technology Delhi, Hauz Khas, New Delhi, India.

d Climate Changes and Data Science Division, CSIR-Indian Institute of Petroleum, Mohkampur, Dehradun 248005, India

e IIT Delhi-Abu Dhabi campus, Khalifa City B, Abu Dhabi 20010, UAE

*Corresponding author's email addresses: haider@iitd.ac.in

tuhin.khan@csir.res.in

	IS	TS	FS	Surfaces
(g)	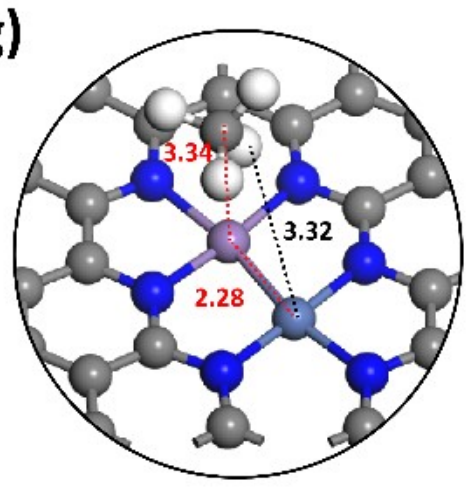	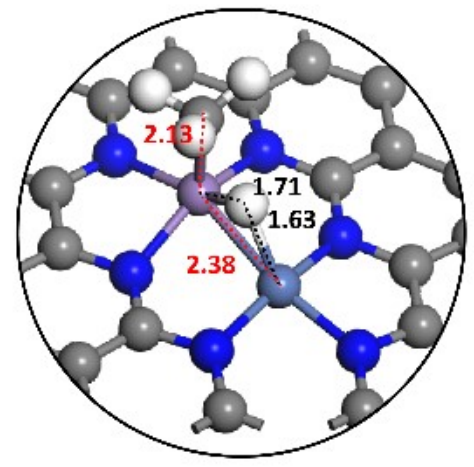	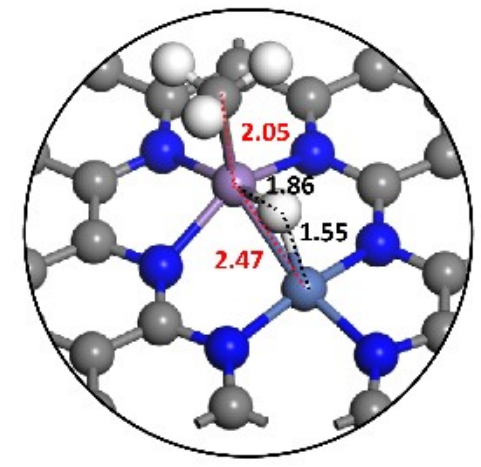	Mn/Ni
(h)	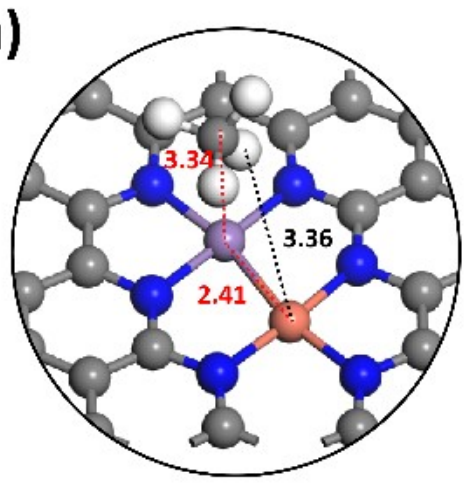	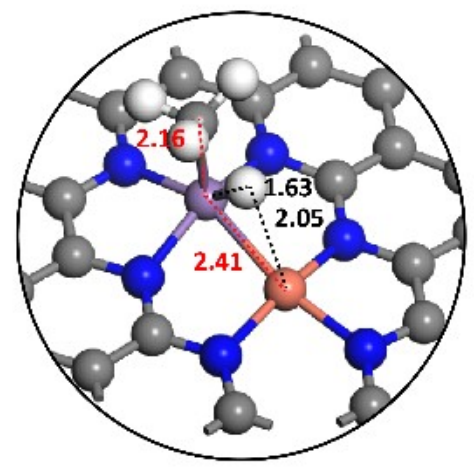	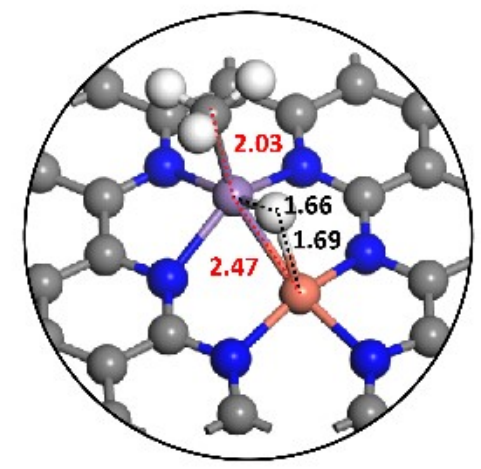	Mn/Cu
(i)	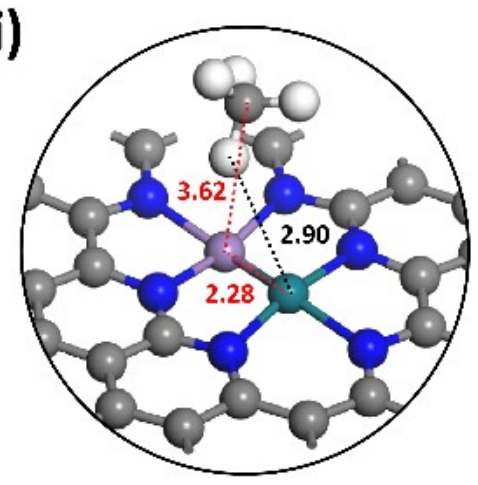	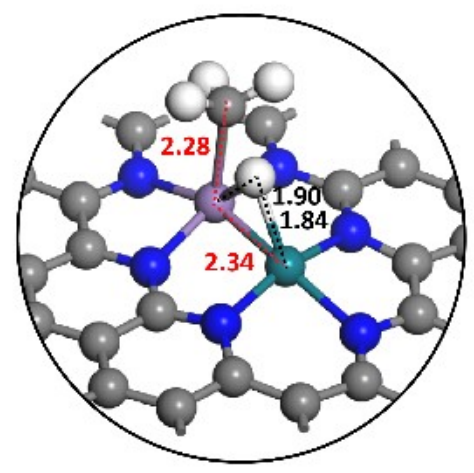	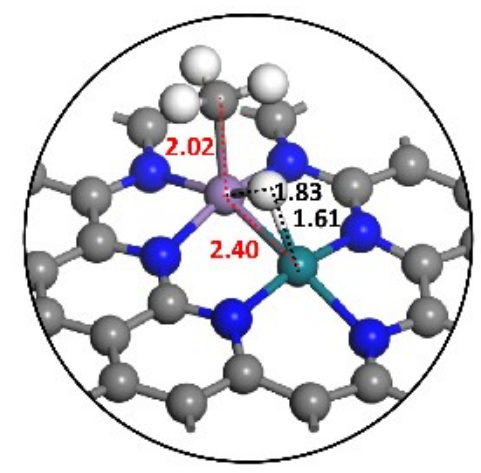	Mn/Rh
(j)	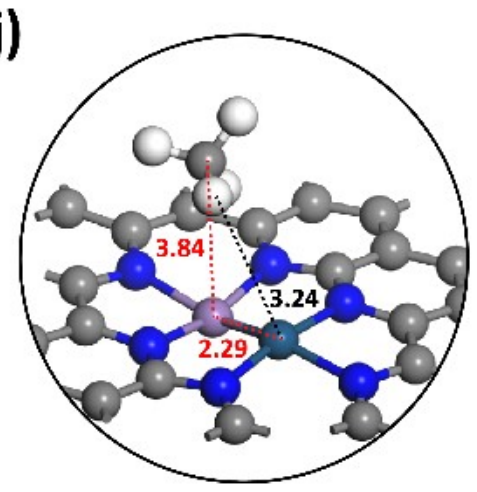	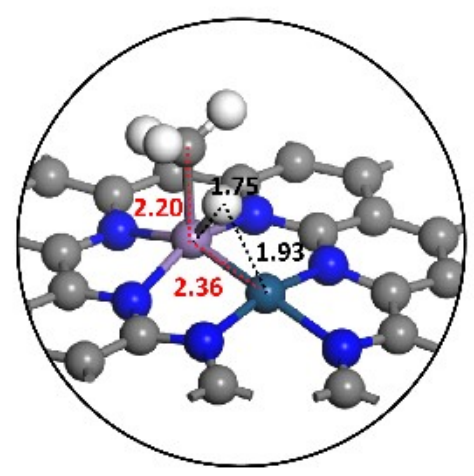	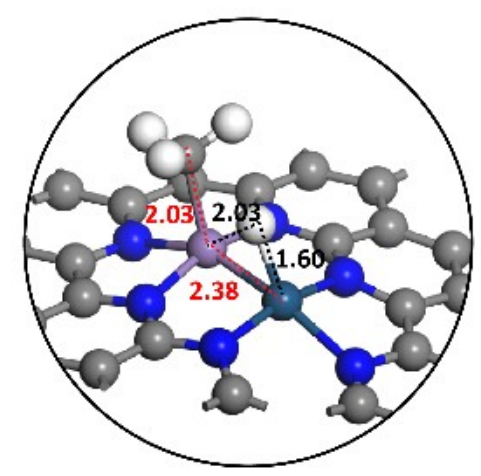	Mn/Ir
(k)	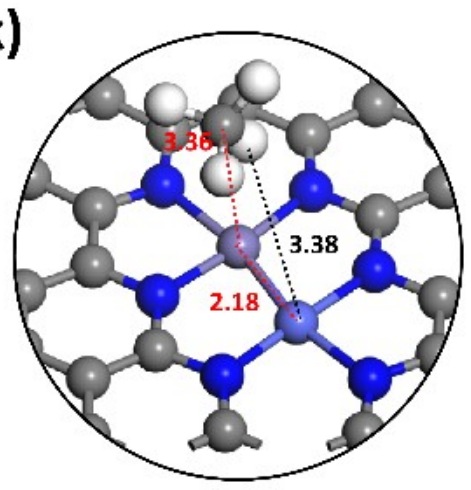	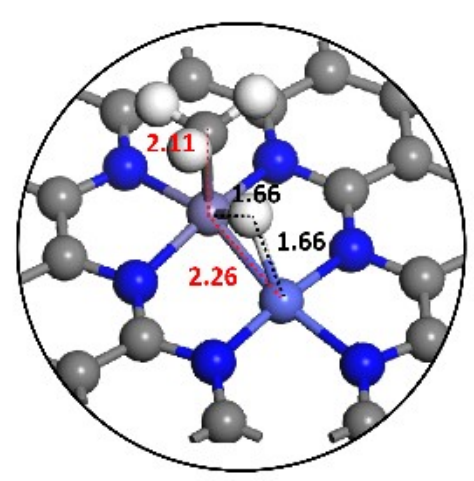	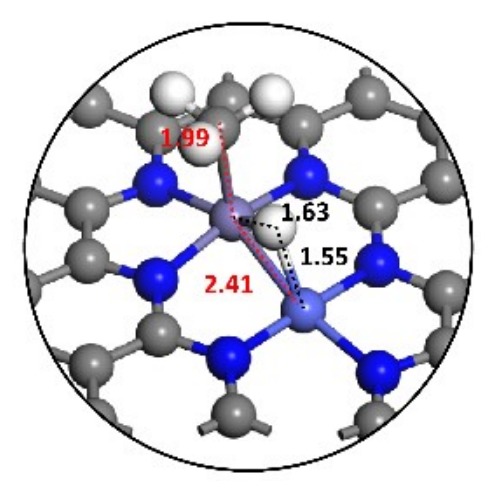	Fe/Co
(l)	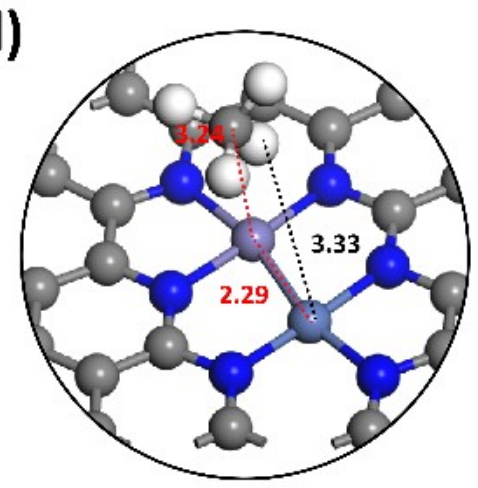	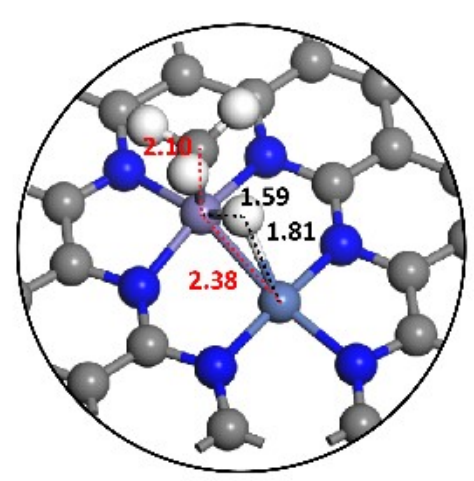	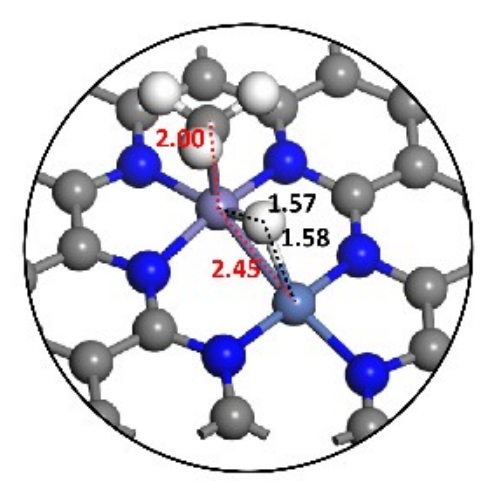	Fe/Ni

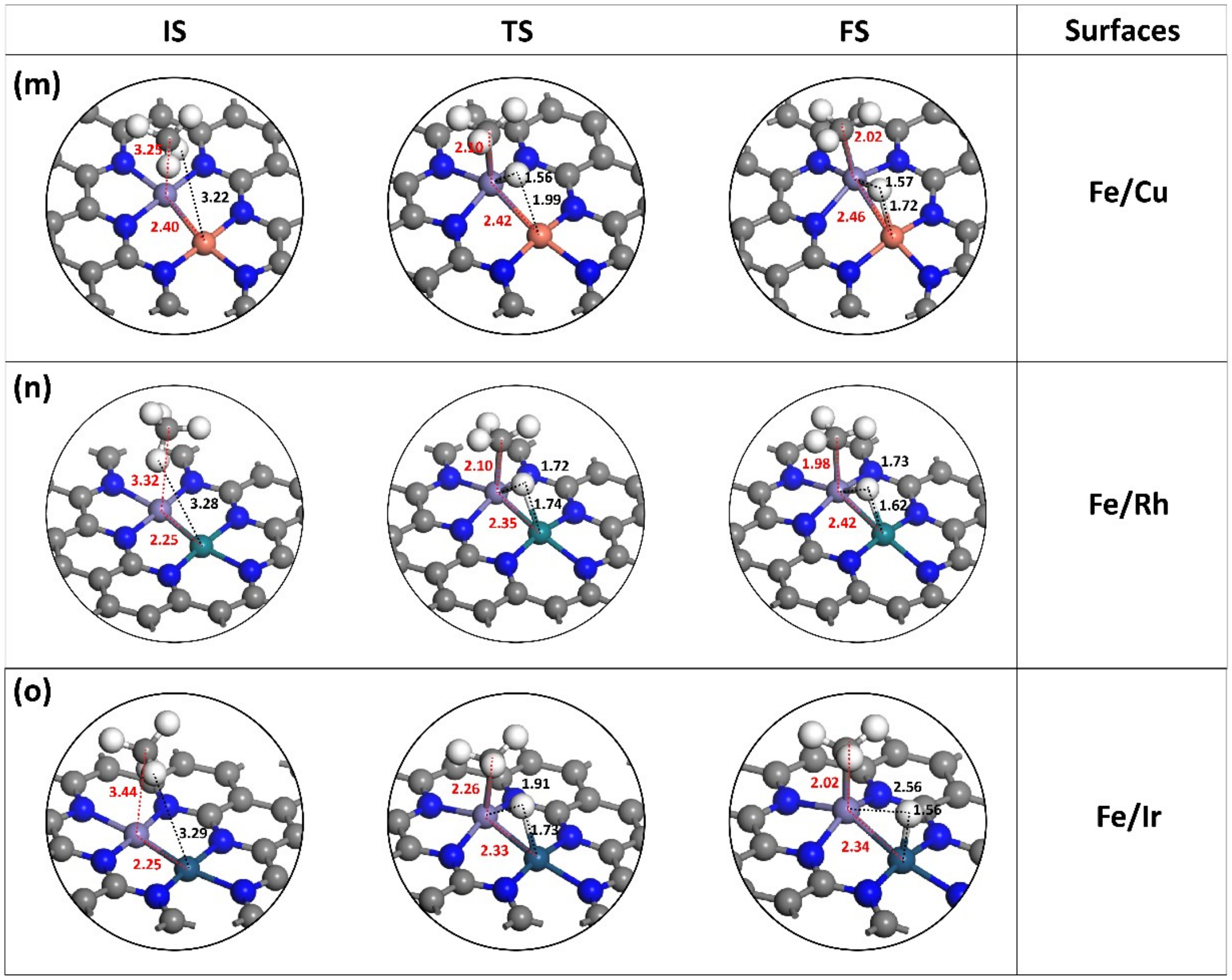

Figure S1. Bond distances for DMSCs built using combinations of M1: (a) Cr, (b) Mn and (c) Fe with the M2 transition metals (Co, Ni and Cu) belonging to the same row in the periodic table.

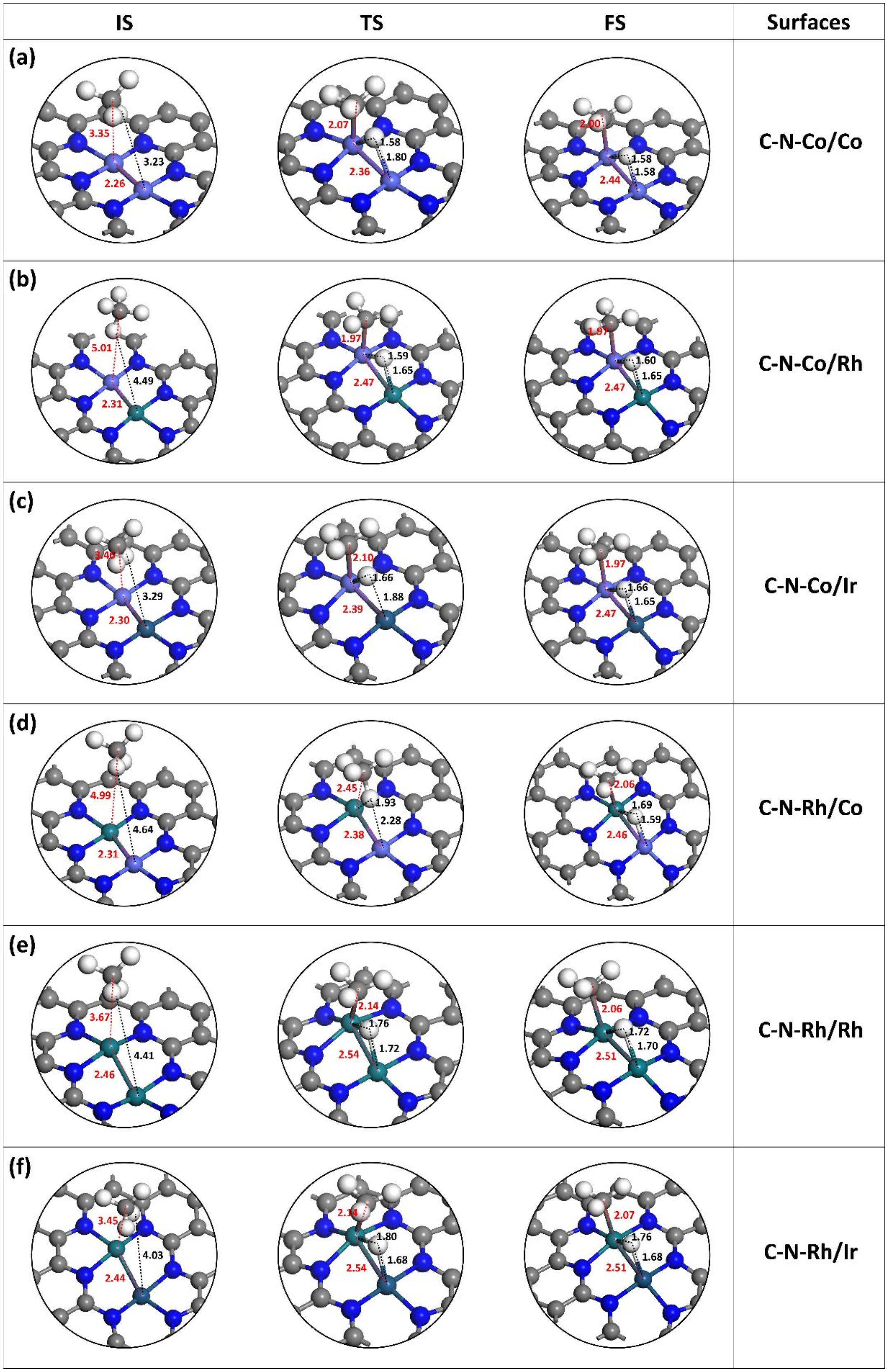
IS
TS
FS
Surfaces
(a)
3.35
3.23
2.26
2.07
1.58
1.80
2.36
2.00
1.58
1.58
2.44
C-N-Co/Co
(b)
5.01
4.49
2.31
1.97
1.59
1.65
2.47
1.97
1.60
1.65
2.47
C-N-Co/Rh
(c)
3.40
3.29
2.30
2.10
1.66
1.88
2.39
1.97
1.66
1.65
2.47
C-N-Co/Ir
(d)
4.99
4.64
2.31
2.45
1.93
2.28
2.38
2.06
1.69
1.59
2.46
C-N-Rh/Co
(e)
3.67
4.41
2.46
2.14
1.76
1.72
2.54
2.06
1.72
1.70
2.51
C-N-Rh/Rh
(f)
3.45
4.03
2.44
2.14
1.80
1.68
2.54
2.07
1.76
1.68
2.51
C-N-Rh/Ir

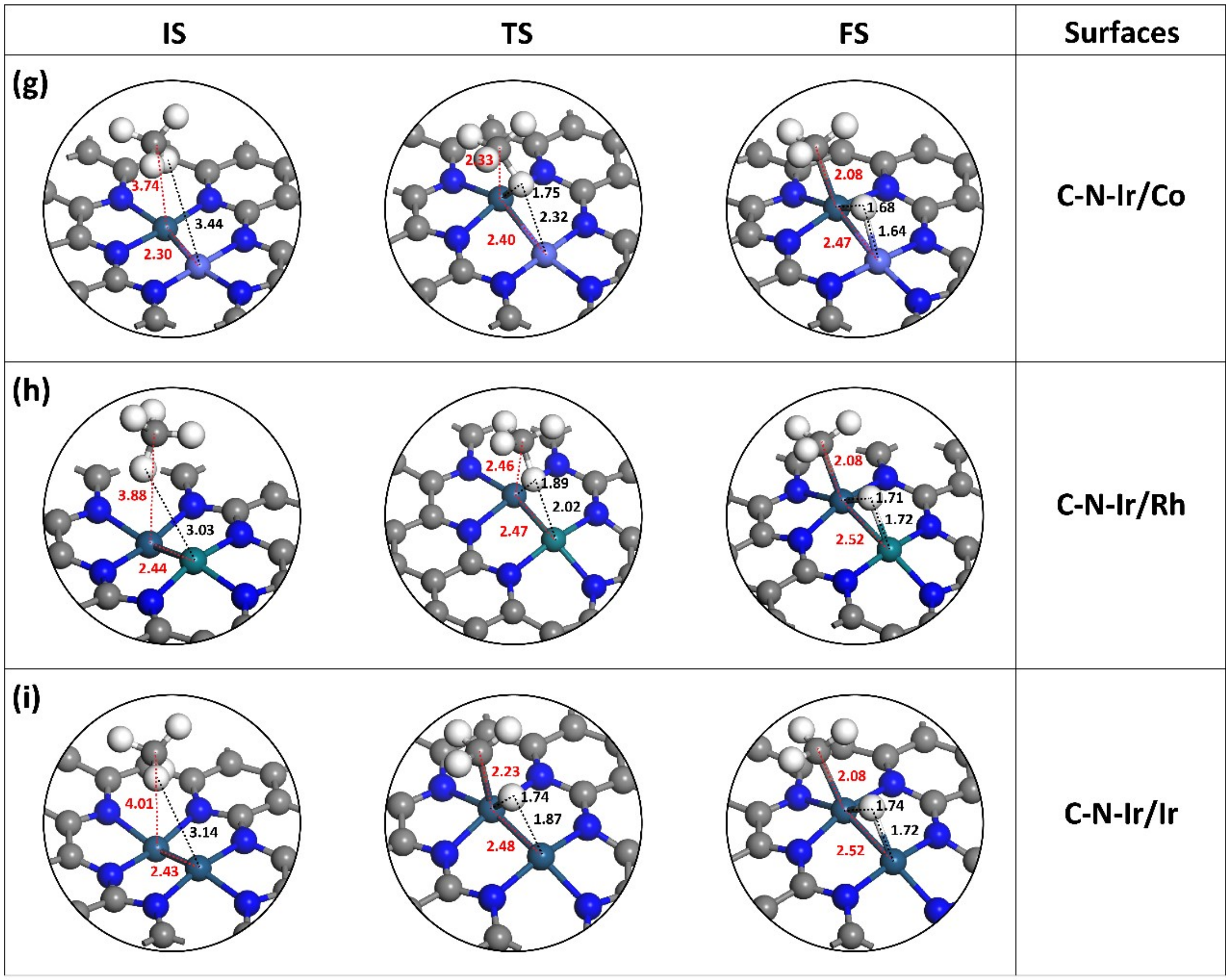

Figure S2. Bond distances for DMSCs built using combinations of M1: (a) Co, (b) Rh and (c) Ir with the M2 transition metals (Co, Rh and Ir) belonging to the same group in the periodic table.

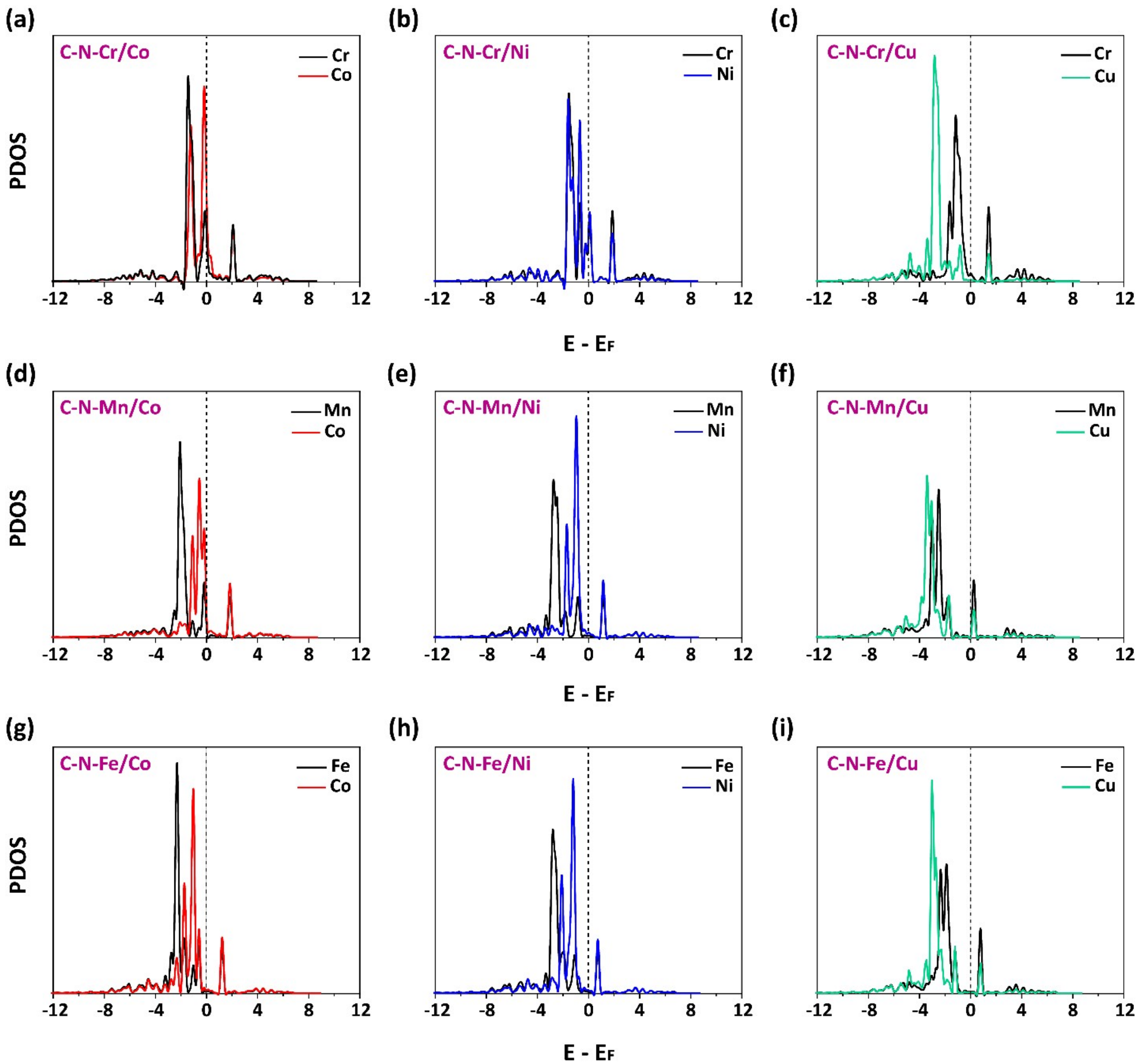

Figure S3. PDOS for DMSCs built using combinations of M1: (a) Cr, (b) Mn and (c) Fe with the M2 transition metals (Co, Ni and Cu) belonging to the same row in the periodic table.

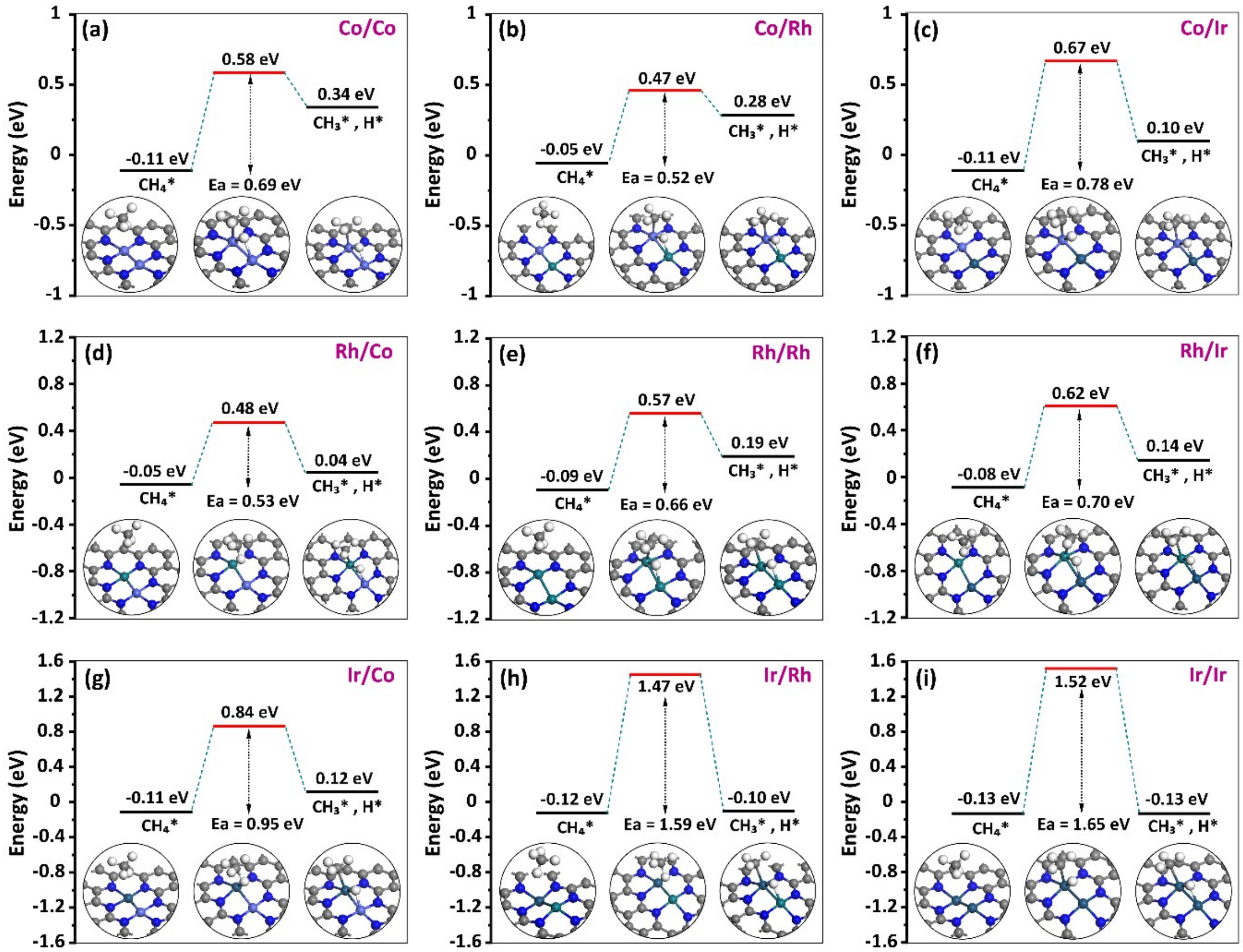

Figure S4(a). C-H bond activation energy trend for methane on DMSCs build using combinations of M1: (a) Co, (b) Rh and (c) Ir with the M2 transition metals (Co, Rh and Ir) belonging to the same group in the periodic table.

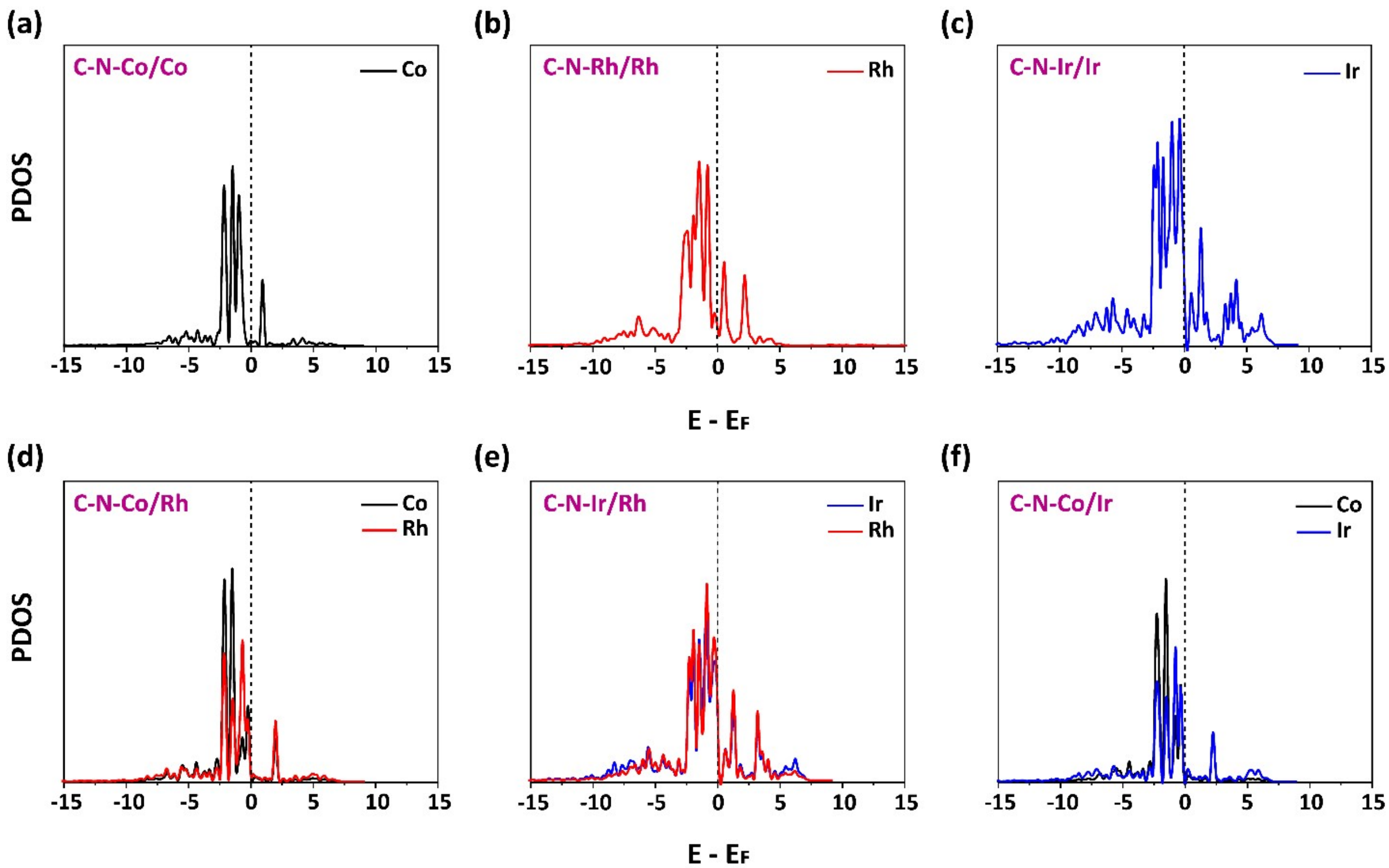

Figure S4(b). PDOS for DMSCs built using combinations of M1: (a) Co, (b) Rh and (c) Ir with the M2 transition metals (Co, Rh and Ir) belonging to the same group in the periodic table.

An investigation of DMSCs with active-site metals varying down the group is also conducted, using group 9 transition metals, namely Co, Rh, and Ir (Figure S4(a)). An increasing Ea is observed in the combinations Rh/Co (0.53 eV) < Rh/Rh (0.66 eV) < Rh/Ir (0.7 eV) and Ir/Co (0.95 eV) < Ir/Rh (1.59 eV) < Ir/Ir (1.65 eV) as the M2 is varied down the group 9 metals. On the contrary, Ea for the DMSCs with Co as M1 first shows a downward trend from Co/Co (0.69 eV) to Co/Rh (0.52 eV), then an upward trend to Co/Ir (0.78 eV). Bond distances have also been reported in Figure S2. Here, the TS M2-H bond distances initially dip and then rise as M2 moves down the group from Co to Rh and then to Ir, given that M1 is constant across the set (Co/Rh/Ir). We observe the same for the 3 sets as Co/Co, Co/Rh, Co/Ir (1.8 A, 1.65 A, 1.88 A); Rh/Co, Rh/Rh, Rh/Ir (2.28 A, 1.72 A, 1.68 A); and Ir/Co, Ir/Rh, Ir/Ir (2.32 A, 2.02 A, 1.87 A). However, if we look at the M1-C distances for the TS for these DMSCs, they decrease from 2.45 A to 2.14 A for Rh/Co to Rh/Ir. In contrast, two contrasting variations emerge for Co/Co, Co/Rh, Co/Ir (a dip from 2.07 A to 1.97 A, followed by an increase to 2.1 A) and Ir/Co, Ir/Rh, Ir/Ir (a rise from 2.33 A to 2.46 A, followed by a dip to 2.23 A). This suggests that a consistent relationship is not directly evident from bond distances. To gain further insights, d-band calculations were performed for M1 and M2, with DOS

representations as shown in Figure S4(b). As Ea increases from Rh/Co (0.53 eV) to Rh/Ir (0.7 eV), the mean d-band centre (ε_m) is detected to first move away from the Fermi level from Rh/Co to Rh/Rh (-1.67 eV to -1.79 eV), then closer for Rh/Ir (-1.36 eV). A similar mismatch is also observed in the case of Ir/Co, Ir/Rh and Ir/Ir DMSCs where the first ε_m shifts towards the Fermi level (-1.79 eV to -1.36 eV) followed by a shift away from fermi level (-1.51 eV) while the Ea increases uniformly from Ir/Co (0.95 eV) to Ir/Ir (1.65 eV). This indicates that ε_m too is not able to capture the variations of Ea with different DMSC configurations.

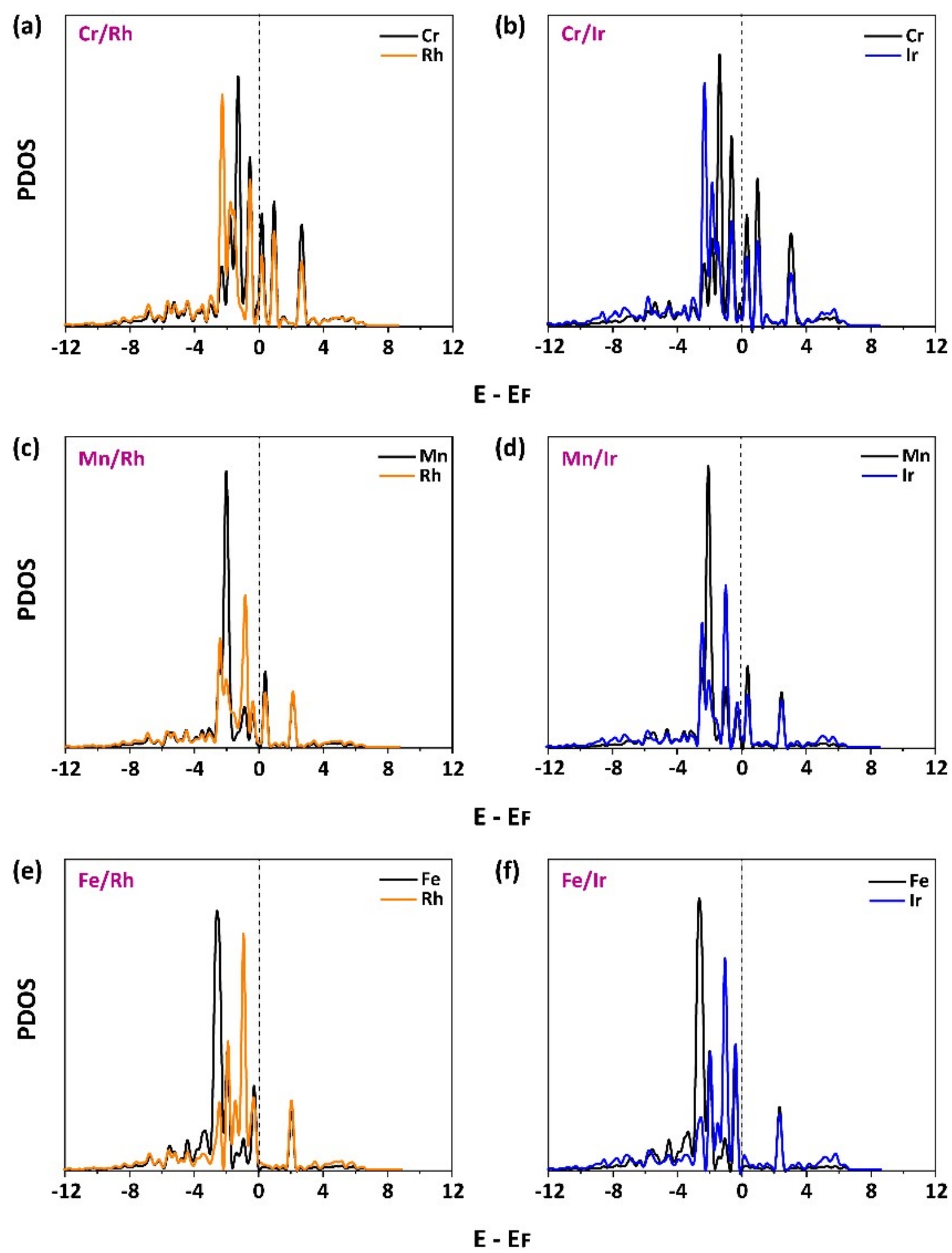

Figure S5. PDOS for DMSCs build using combinations of M1: (a) Cr, (b) Mn and (c) Fe with the M2 transition metals (Rh and Ir) belonging to the same group in the periodic table.

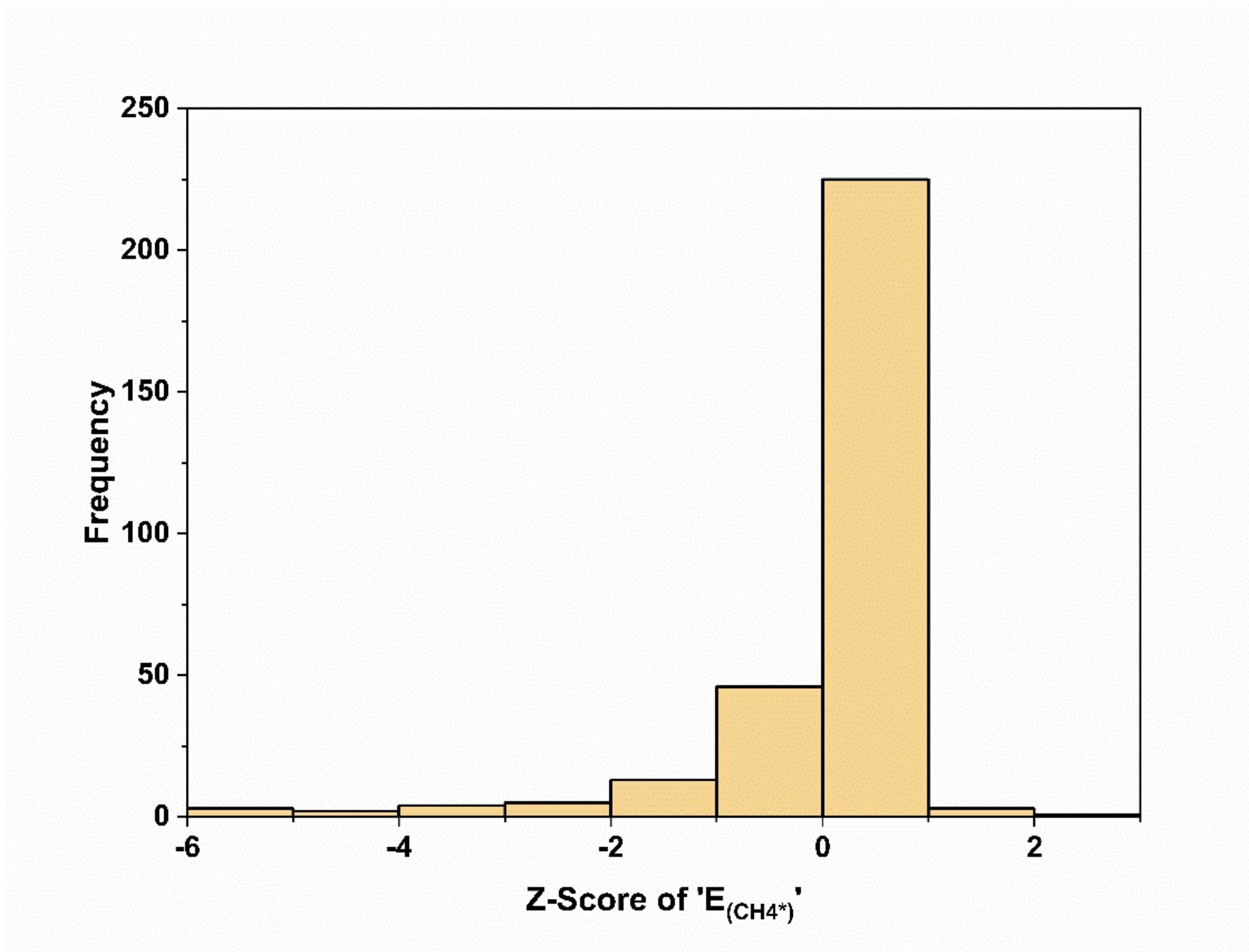

Figure S6(a). Distribution of $E_{(CH4*)}$ values based on their Z-score.

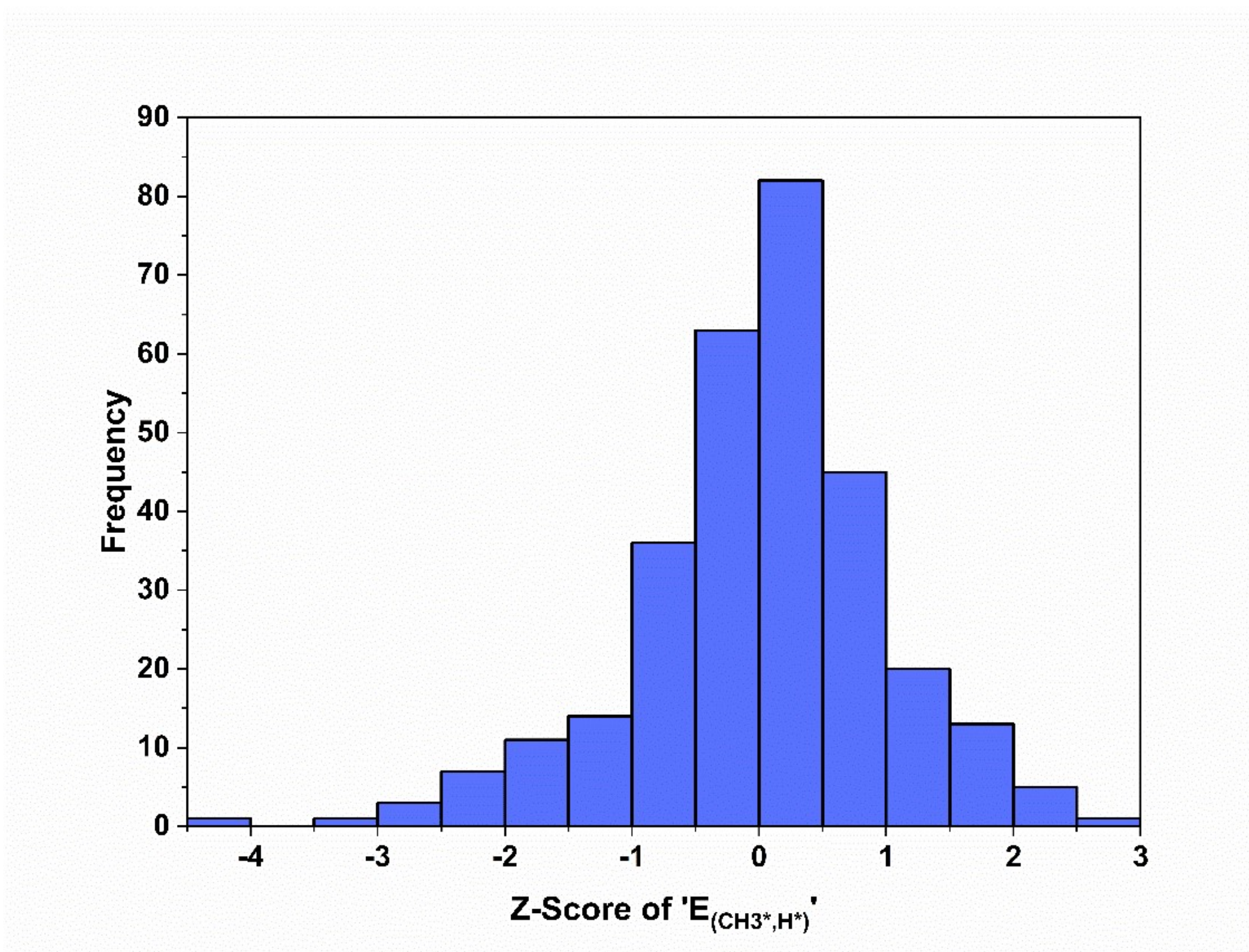

Figure S6(b). Distribution of E(CH3*, H*) values based on their Z-score.

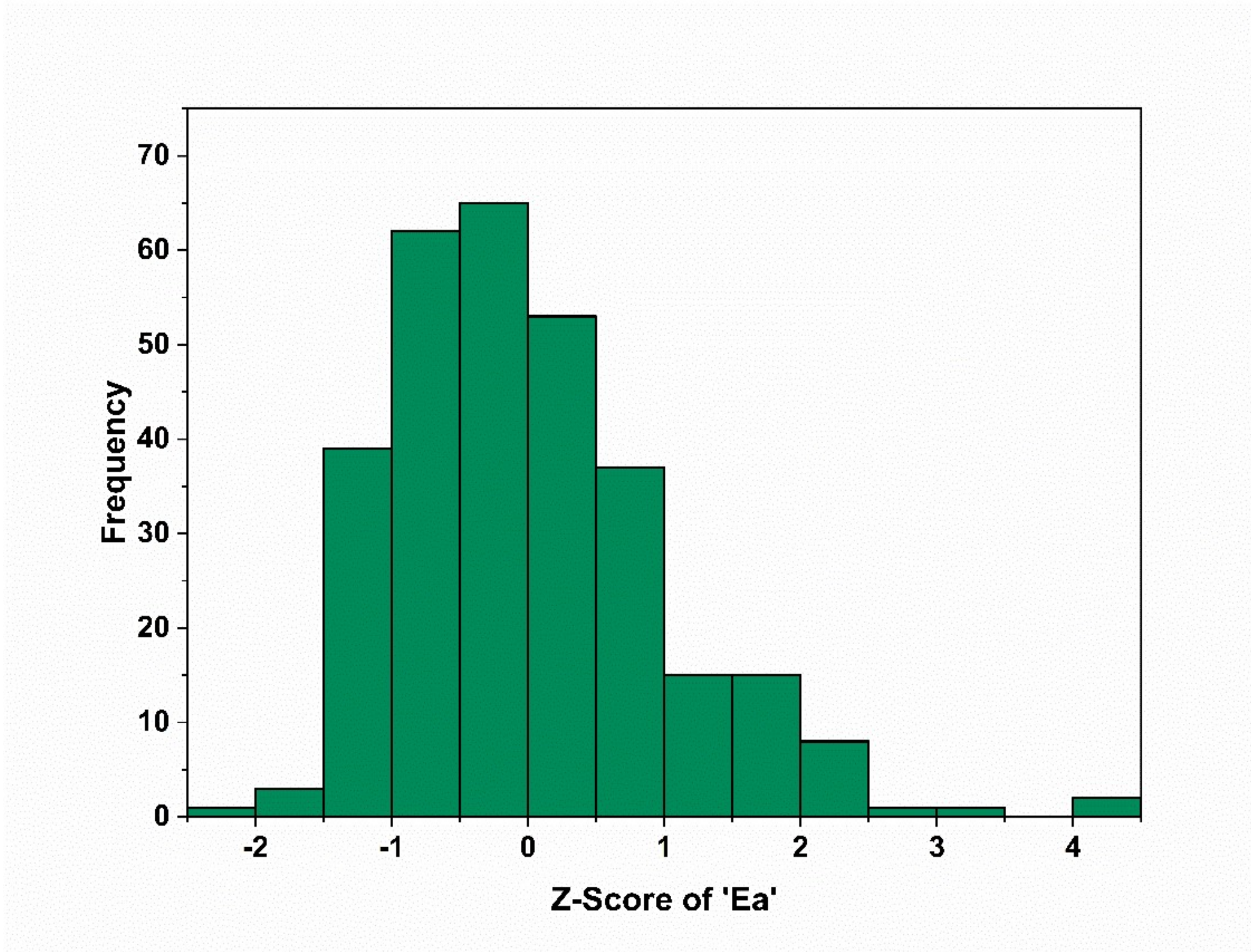

Figure S6(c). Distribution of Ea values based on their Z-score.

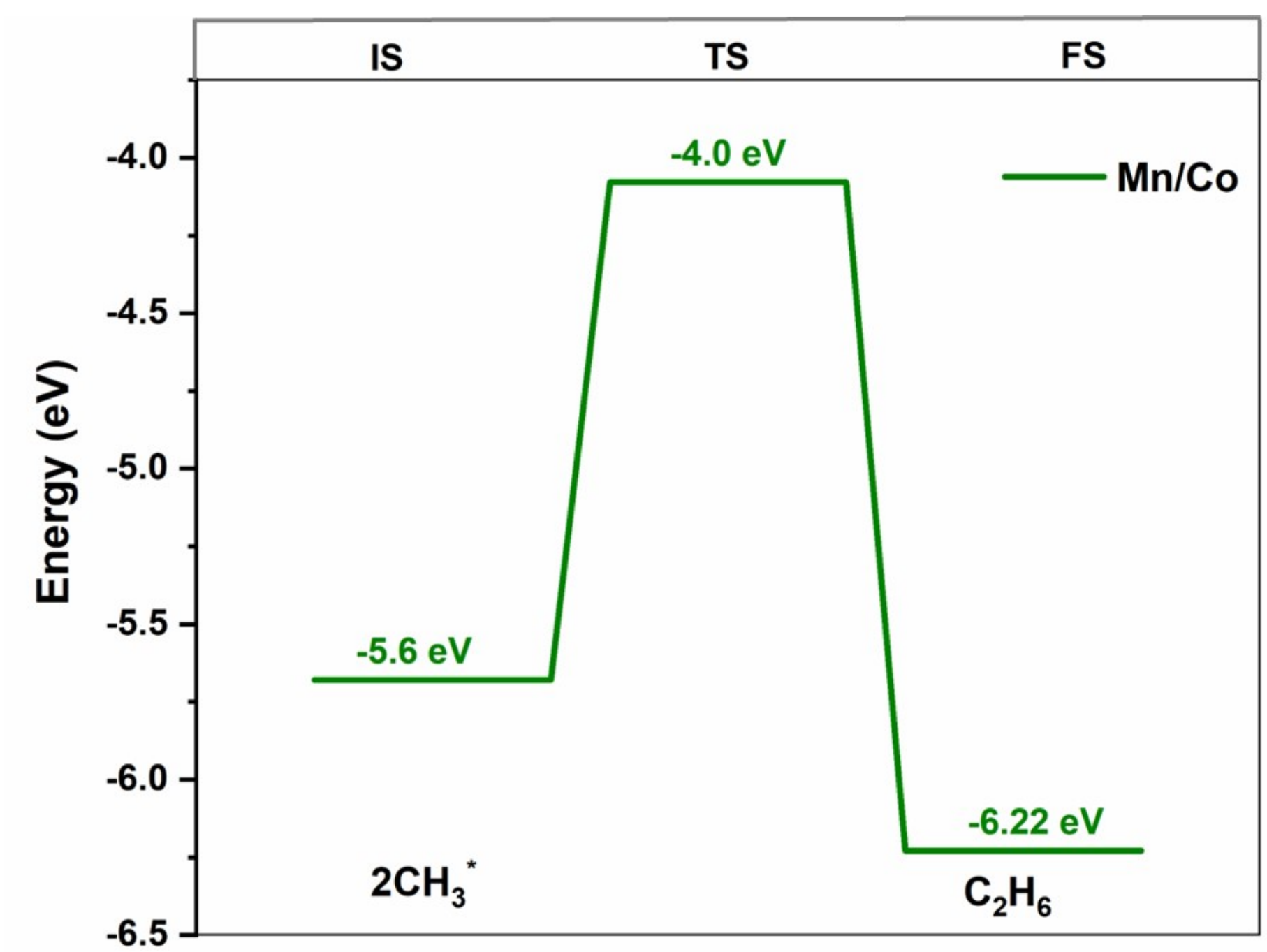

Figure S7. Energy change profile depicting the C-C coupling reaction for Mn/Co based system.

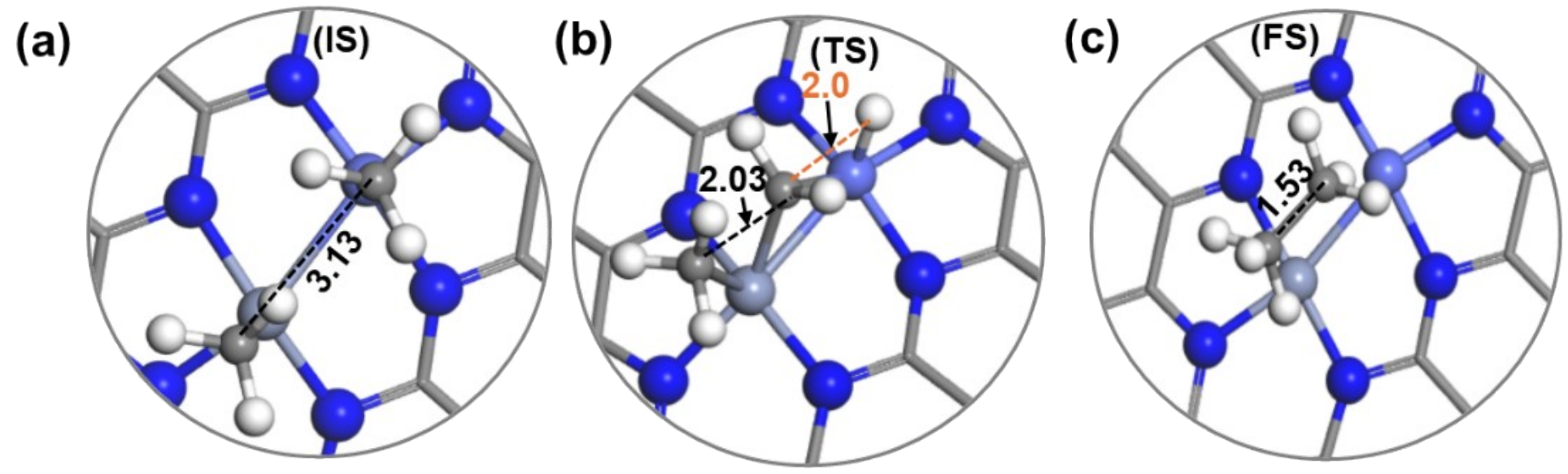

Figure S8. Initial state (IS), transition state (TS), and final state (FS) structures for the Cr/Cu system during the C-C coupling reaction.

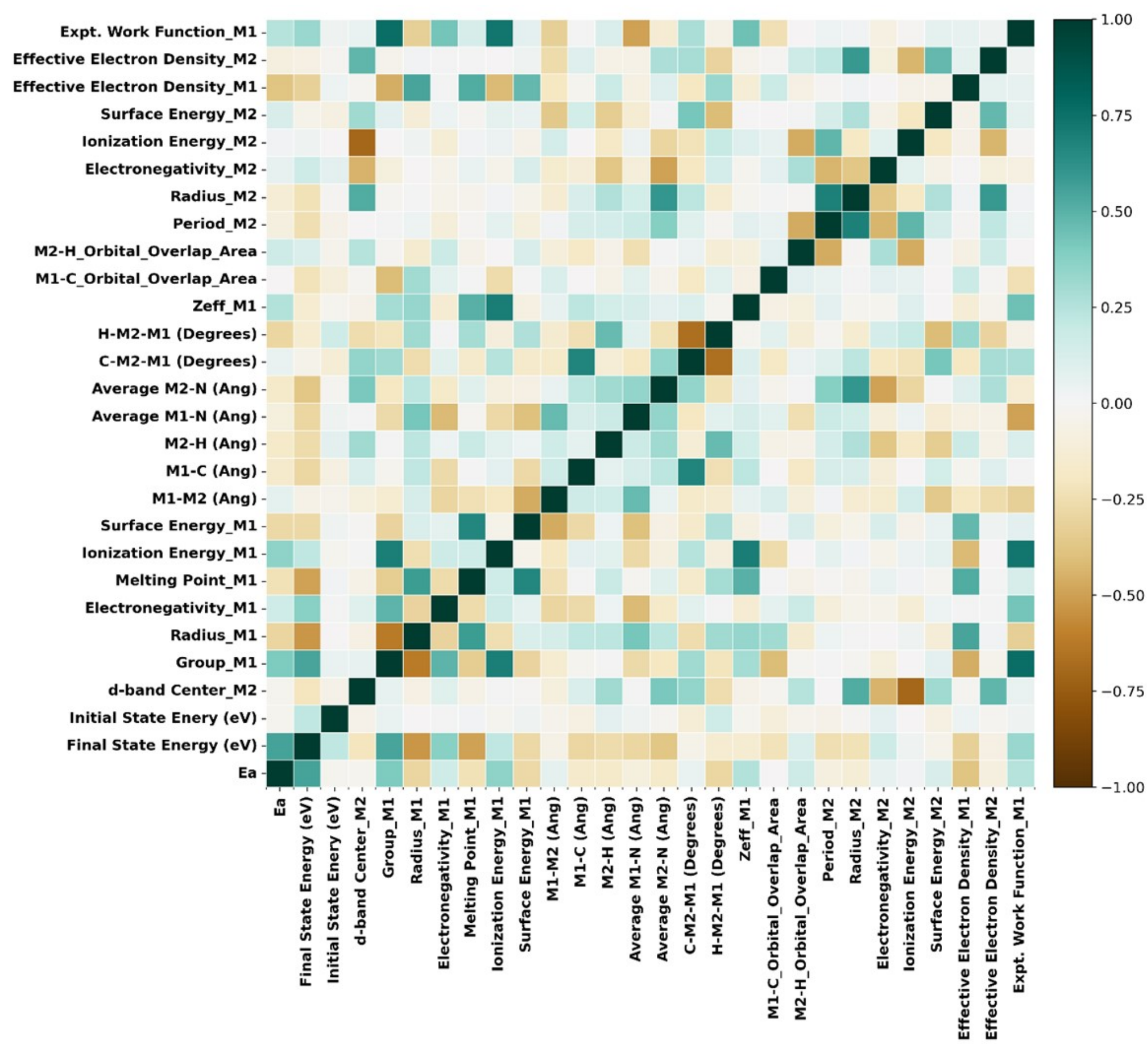

Figure S9. Pearson correlation plot depicting linear dependency between feature space (27 features) and target space, for E_a.

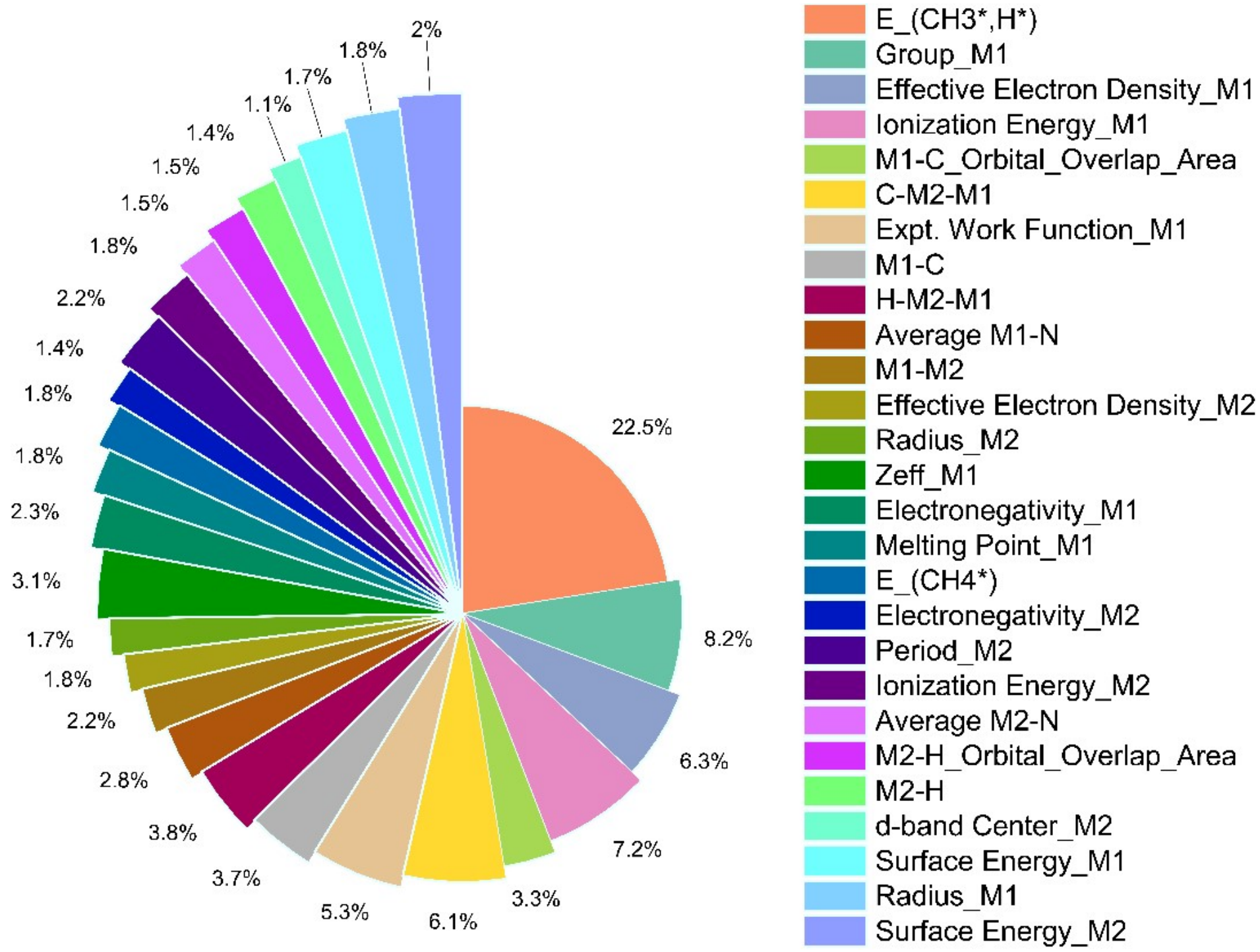

Figure S10. Feature importance for E_a prediction using ETR model.

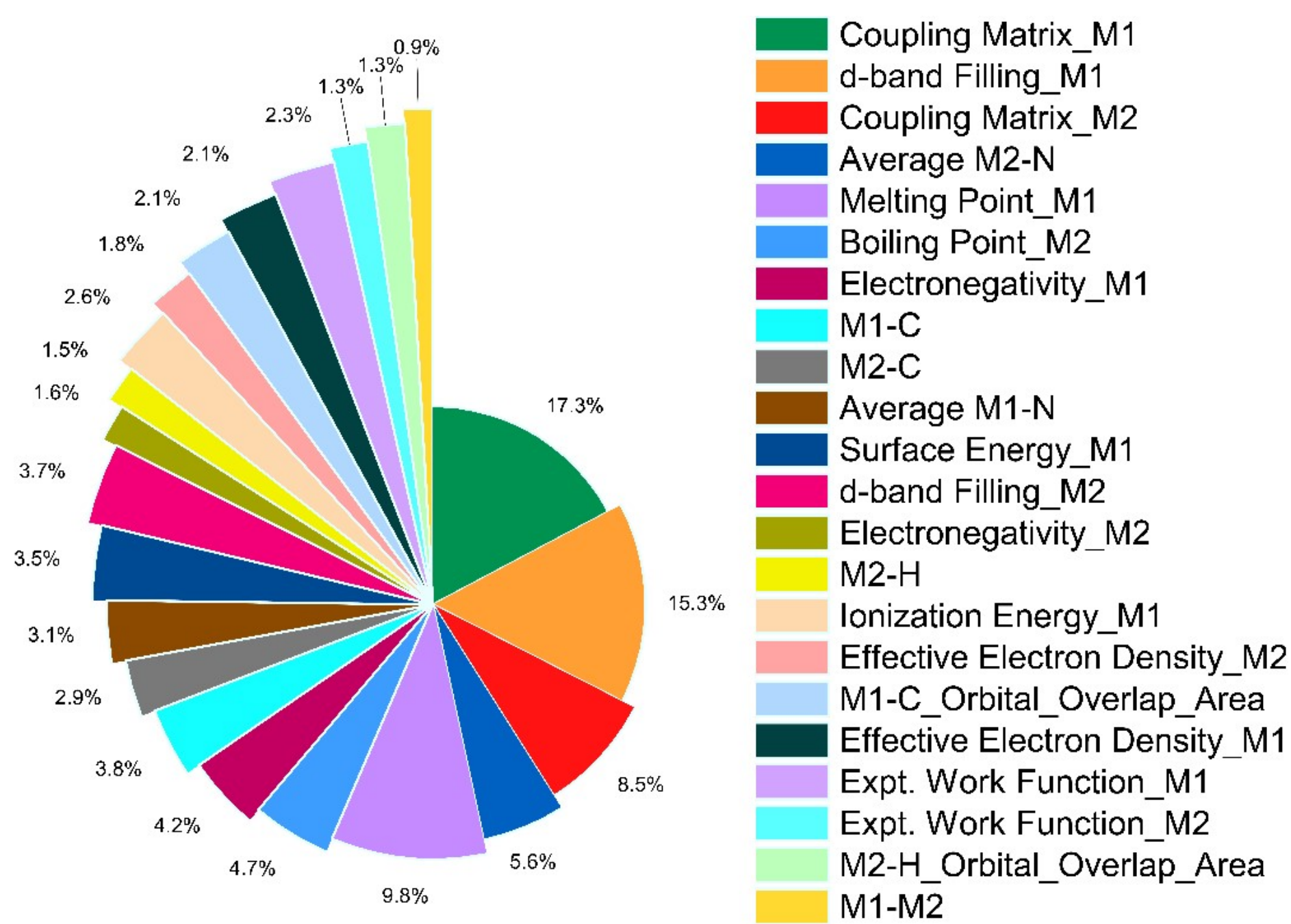

Figure S11. Feature importance for $E_{(CH3*, H*)}$ prediction using the ETR model.

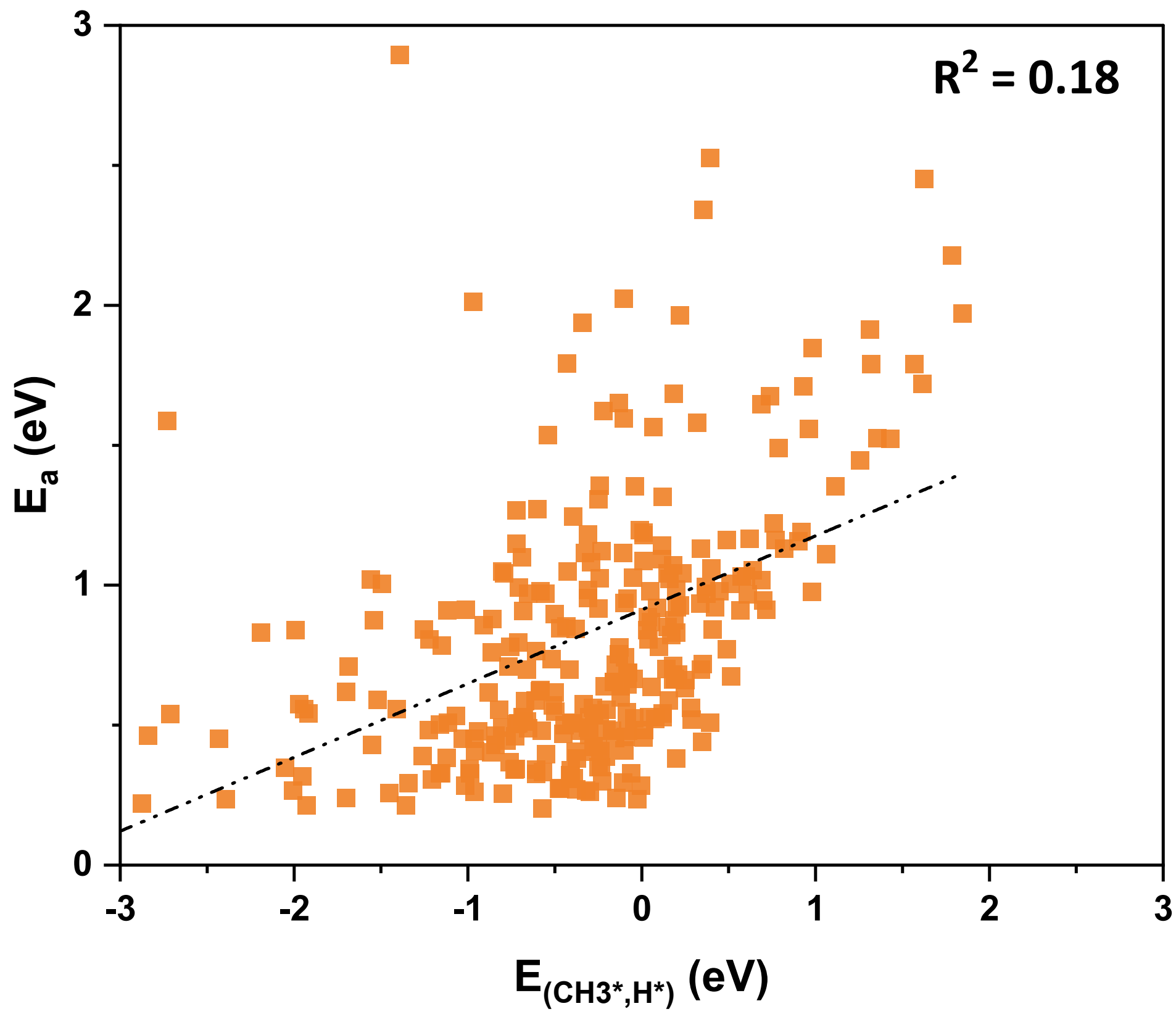

Figure S12. Brønsted–Evans–Polanyi (BEP) scaling relationship between the C-H activation energy (E_a) and Final State Adsorption Energy ($E_{(CH3*, H*)}$).

(a)

(b)

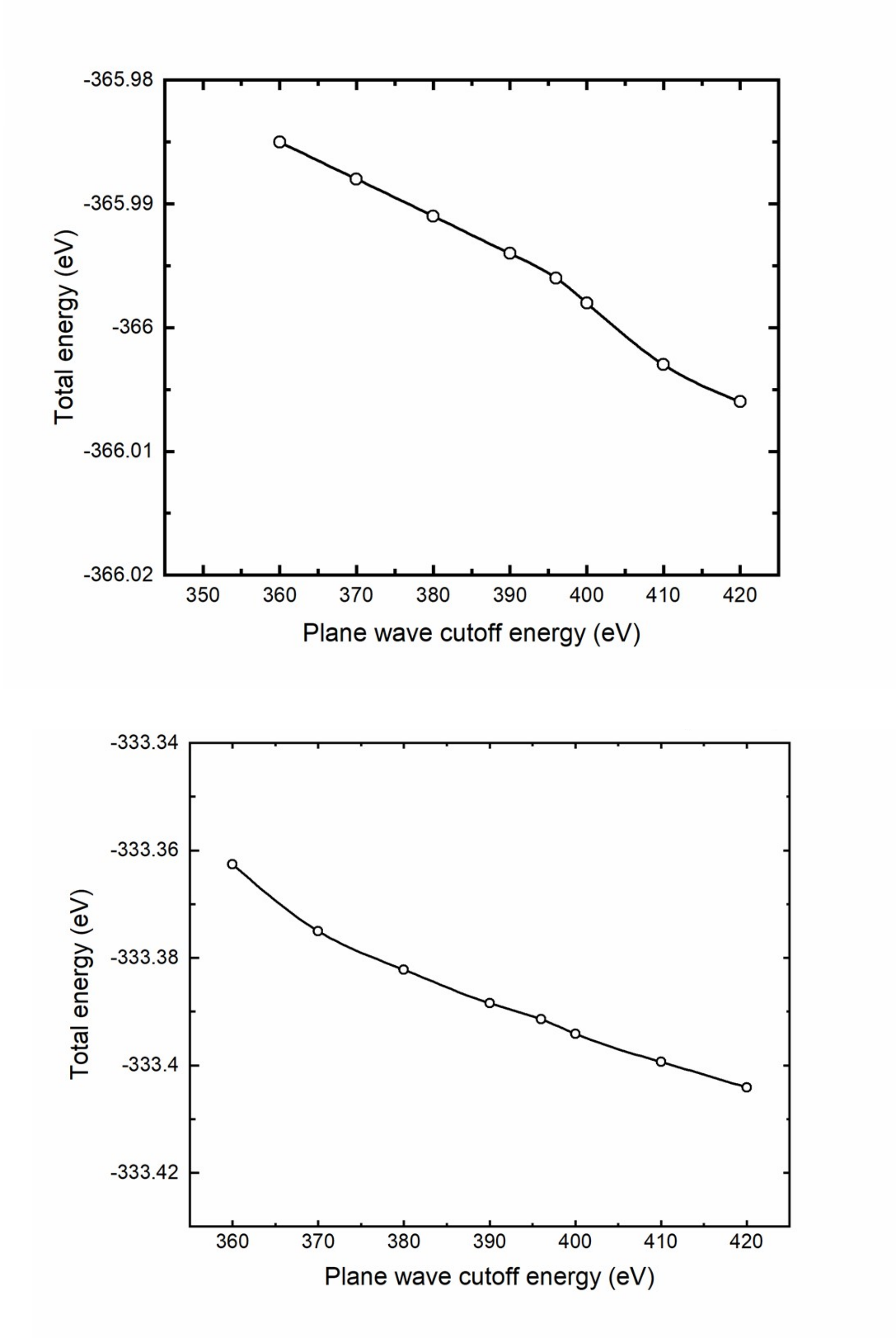

Figure S13. Convergence of the extrapolated total energy with respect to the plane-wave cutoff energy for (a) graphene and (b) the representative Fe-Fe/N-doped graphene catalyst model. All calculations were performed using fixed optimised geometries and a $2 \times 2 \times 1$ k-point mesh.

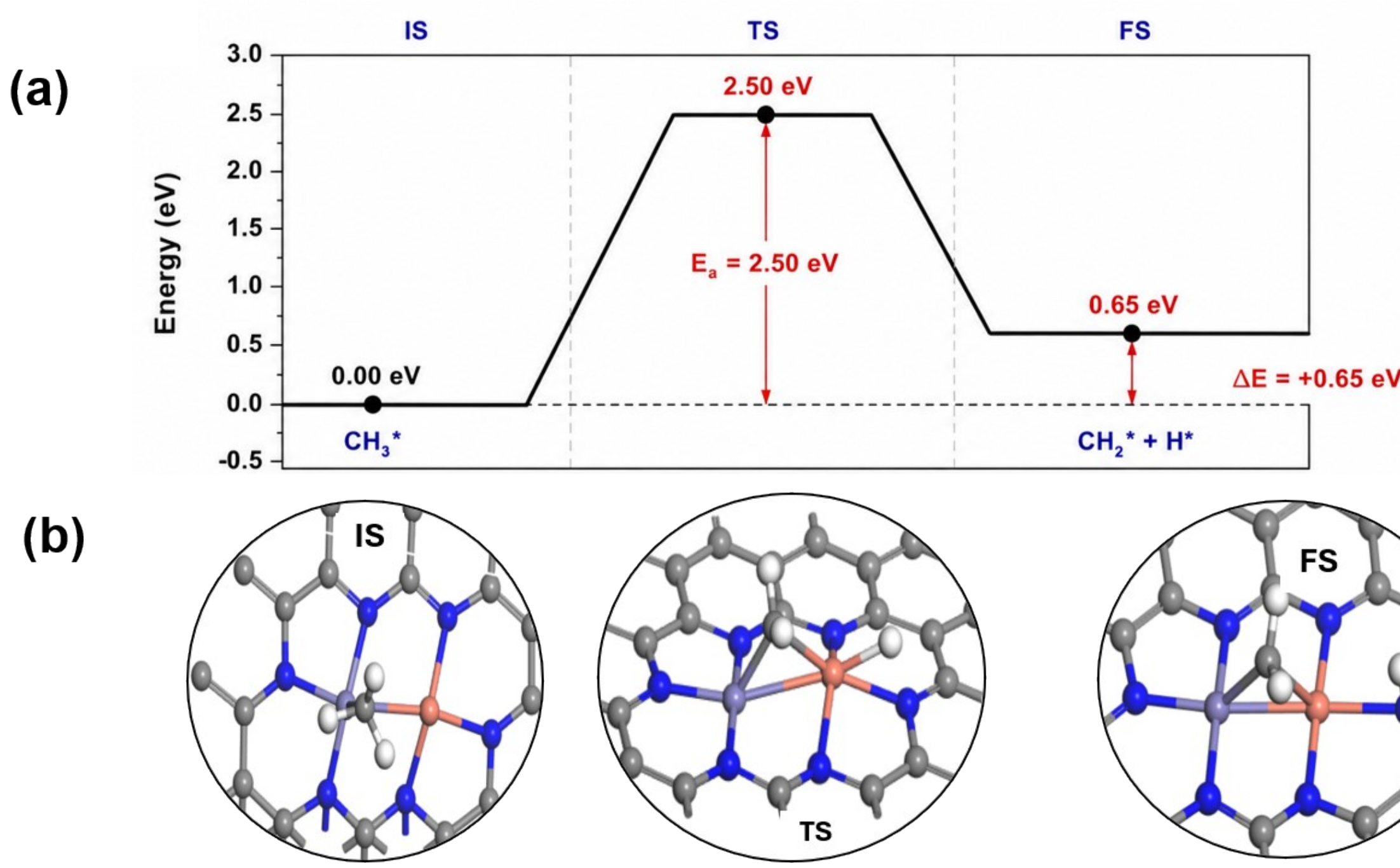

Figure S14. (a) Energy profile for the CH_3 dehydrogenation pathway $CH_3^* \rightarrow CH_2^* + H^*$) on the representative Fe/Cu based system. **(b)** Optimised geometries of the initial state (IS), transition state (TS), and final state (FS) corresponding to the CH_3 dehydrogenation reaction.

Table S1. The range of hyperparameters used in each ML model.

Models	Hyperparameter	Values
**KNN**	number of nearest neighbors	2, 3, 4, 5, 6, 7, 8, 9, 10
	weights	uniform, distance
**SVR**	kernel	radial basis function
	kernel coefficient (gamma)	0.1, 0.01, 0.001, 0.0001, 0.00001
	regularization parameter (C)	1, 10, 100, 1000, 10000, 100000
**RFR**	number of decision trees	50, 100, 200, 300, 400, 500, 600, 700, 800
	maximum depth of the tree	2, 3, 4, 5, 6, 7, 8
**ETR**	number of decision trees	50, 100, 200, 300, 400, 500, 600, 700, 800
	maximum depth of the tree	2, 3, 4, 5, 6, 7, 8
**GBR**	number of decision trees	50, 100, 200, 300, 400, 500, 600, 700, 800
	learning rate	0.1, 0.3, 0.4, 0.5, 0.6, 0.7, 0.8
	maximum depth of the tree	2, 3, 4, 5, 6, 7, 8
**ANN**	activation function	hyperbolic tangent, rectified linear unit
	loss function	mean square error, mean absolute error
	optimizer	stochastic gradient descent, root mean square propagation

Table S2: (a) ENCUT convergence for graphene at $2 \times 2 \times 1$ k-point mesh.

ENCUT (eV)	Total energy, $E_{\sigma\to 0}$ (eV)	Consecutive energy difference, |ΔE| (eV)
360	-365.98585757	-
370	-365.98867055	0.00281298
380	-365.99112293	0.00245238
390	-365.99401505	0.00289212
**396**	**-365.99615591**	**0.00214086**
400	-365.99817151	0.00201560
410	-366.00298294	0.00481143
420	-366.00797691	0.00499397

Note: |ΔE| is the absolute energy difference between the current cutoff and the immediately preceding cutoff. The 396 eV row is shown in bold to identify the selected production cutoff.

(b) ENCUT convergence for Fe-Fe/N-doped graphene at $2 \times 2 \times 1$ k-point mesh.

ENCUT (eV)	Total energy, Eσ→0 (eV)	Consecutive energy difference, |ΔE| (eV)
360	-333.36255430	-
370	-333.37502812	0.01247382
380	-333.38217133	0.00714321
390	-333.38845256	0.00628123
**396**	**-333.39145418**	**0.00300162**
400	-333.39411525	0.00266107
410	-333.39937999	0.00526474
420	-333.40410883	0.00472884

Note: |ΔE| is the absolute energy difference between the current cutoff and the immediately preceding cutoff. The 396 eV row is shown in bold to identify the selected production cutoff.

Table S3: K-point convergence of the Fe-Fe/N-doped graphene model at ENCUT = 396 eV

K-point mesh	Total energy, Eσ→0 (eV)	ΔE_k Difference from $5 \times 5 \times 1$ (eV)
1×1×1	-333.47879350	0.07770193
2×2×1	**-333.39145418**	**0.00963739**
3×3×1	-333.39324861	0.00784296
4×4×1	-333.39382620	0.00726537
5×5×1	--333.40109157	0.00000000

Note: $\Delta E_k$is the absolute energy difference relative to the $5 \times 5 \times 1$k-point mesh. The $2 \times 2 \times 1$ row is shown in bold to identify the selected mesh.

Vibrational analysis of transition states. Finite-difference vibrational frequency calculations were performed for selected transition states associated with the C-C coupling and first C-H activation transition states used in the direct comparison between the DFT and machine-learning results. For each validated transition state, the calculated frequency spectrum and corresponding normal-mode eigenvectors were examined. Each accepted structure exhibited one significant imaginary frequency associated with the intended reaction coordinate, while the remaining chemically meaningful vibrational modes were real. The calculated frequencies are summarised in Table S4.

Table S4. Vibrational validation of representative transition states involved in C-C coupling and C-H activation on dual-metal-site catalysts.

Reaction step	***Catalyst***	***Imaginary frequency (cm^{-1})***	***Assignment of the imaginary mode***
**C-C coupling**	Fe-Ni	488.26	C-C bond formation between the two CH_3 fragments
	Fe-Co	697.49	C-C bond formation between the two CH_3 fragments
	Fe-Cu	546.39	C-C bond formation between the two CH_3 fragments
	Fe-Fe	597.12	C-C bond formation between the two CH_3 fragments

	Cr-Cu	623.70	C-C bond formation between the two CH_3 fragments
	Cr-Ni	1010.02	C-C bond formation between the two CH_3 fragments
	Cr-Co	609.51	C-C bond formation between the two CH_3 fragments
	Cr-Ir	591.27	C-C bond formation between the two CH_3 fragments
	Cr-Rh	221.87	C-C bond formation between the two CH_3 fragments
	Mn-Co	542.15	C-C bond formation between the two CH_3 fragments
**First C-H activation**	Fe-Fe	502.47	C-H bond cleavage and transfer of H toward the adjacent active site
	Fe-Co	819.41	C-H bond cleavage and transfer of H toward the adjacent active site
	Fe-Ni	1305.45	C-H bond cleavage and transfer of H toward the adjacent active site

Table S5. Comparison of CH_3 dehydrogenation and CH_3-CH_3 coupling barriers on representative dual metal-site catalysts**.**

**Catalyst**	**Ea, CH_3 dehydrogenation (eV)**	**Ea, CH_3-CH_3 coupling (eV)**	**Kinetically preferred pathway**

Fe-Cu	2.50	0.70	CH_3-CH_3 coupling
Cr-Ni	1.77	1.30	CH_3-CH_3 coupling
Fe-Ni	1.65	0.50	CH_3-CH_3 coupling
